# Entrenchment of germline amino-acid differences in antibody affinity maturation

**DOI:** 10.64898/2026.04.21.720000

**Published:** 2026-04-23

**Authors:** Noam Harel, Kevin Sung, Will Dumm, Mackenzie M. Johnson, David Rich, Julia Fukuyama, Hugh K. Haddox, Frederick A. Matsen

**Affiliations:** 1 Computational Biology Program, Fred Hutchinson Cancer Center, Seattle, WA, USA; 2 Department of Statistics, Indiana University, Bloomington, IN, USA; 3 Department of Genome Sciences, University of Washington, Seattle, WA, USA; 4 Department of Statistics, University of Washington, Seattle, WA, USA; 5 Howard Hughes Medical Institute, Seattle, WA, USA

## Abstract

Entrenchment — epistasis that locks in amino acid differences between homologous proteins, so each disfavors substitutions toward the other’s state — has been demonstrated along individual protein lineages over deep evolutionary time. Antibodies offer a unique system for studying entrenchment: multiple homologous germline V gene paralogs provide diverse starting points, and the rapid somatic evolution of affinity maturation generates dense phylogenies from which selection on germlineencoded residues can be inferred. Using DASM, a deep learning model that separates selection from mutation in antibody repertoire data, we test for entrenchment across immunoglobulin heavy chain variable (IGHV) genes. We detect entrenchment at two levels of germline divergence, driven by different sources of epistasis. Within V gene families (up to ~20% amino acid divergence), entrenched sites cluster at the borders of the complementarity-determining regions (CDRs, the antigen-binding loops) and show high germline diversity. These sites contact antigen, light chain, and the heavy chain CDR3 loop, all of which are encoded independently of the IGHV germline. This pattern is consistent with epistasis from genetically uncoupled partners. Between V gene families, at deeper levels of divergence (25–40%), entrenchment additionally includes positions in the framework scaffold distant from binding interfaces, suggesting a larger contribution from intra-heavy-chain structural constraints. Observed mutation frequencies in human repertoires corroborate these predictions where data are sufficient. Together, these results demonstrate that the rapid somatic evolution of antibodies can serve as a lens for revealing epistatic constraints acting on germline-encoded residues, including constraints imposed by genetically uncoupled partners assembled during B cell development.

## Introduction

Amino acid differences between homologous proteins can become entrenched: maintained by epistatic constraints such that each homolog disfavors substitutions toward the other’s state [1]. Previous studies have attributed entrenchment to intra-protein epistasis accumulating along single lineages over deep evolutionary time. For example, experimental studies found that restrictive mutations in steroid hormone receptors created an “epistatic ratchet” preventing reversal [2], and deep mutational scanning of Hsp90 across reconstructed ancestors showed that mutational effects shift pervasively over a billion years of evolution [3]. Consistent with these observations, computational studies showed that entrenchment can arise under purifying selection, as substitutions become contingent on their predecessors and increasingly costly to revert [1, 4]. Beyond these single-lineage comparisons, deep mutational scanning of HIV Env variants was used to quantify levels of entrenchment between two substantially diverged co-existing homologs [5].

**Figure 1:**
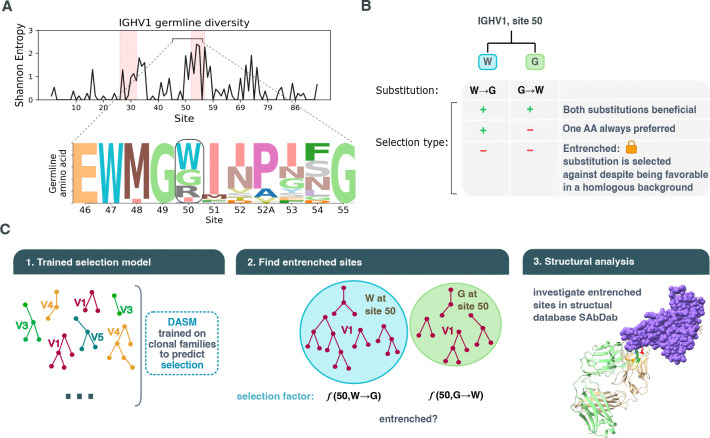
Detecting entrenchment at germline divergent sites using DASM selection factors. **(A)** When V genes have different germline amino acids at homologous positions, epistatic interactions can cause substitutions toward the other V gene’s germline state to be strongly selected against in each genetic background. Examining entropy across sites reveals regions with germline diversity; entrenchment analysis requires such diversity at a site. We select site 50, which has germline diversity, as an example for (B); in this example it contains a Tryptophan (W), a Glycine (G), an Arginine (R) or other less common options, depending on the V gene. **(B)** To test entrenchment between every two options, for example Tryptophan and Glycine, we compare the selection on a germline W mutating to G versus a germline G mutating to W. Three outcomes are possible: diversifying selection at the site (substitutions in both directions are favored), directional selection (one amino acid is always preferred), or entrenchment, where both reciprocal substitutions are disfavored, indicating the site resists change toward its homologous state. **(C)** Approach: we use a DASM model trained on clonal families across all V family types to predict selection factors per site, running inference on a held-out test dataset. Sequences are divided by their germline amino acid at the site of interest, if unmutated at that site. We then examine what DASM predicts as the selection factor at the site: in this site 50 example for W→G in one group and G→W in the other.

Antibodies offer an unusually powerful system for studying entrenchment at multiple evolutionary scales. The human genome encodes approximately 50 functional immunoglobulin heavy chain variable (IGHV) genes at a single locus, all paralogs of a common ancestor [6]. These paralogs are conventionally grouped by sequence similarity into seven families (IGHV1–IGHV7) [7], and have diversified along the germline to now differ at up to ~40% of amino acid positions (Figure 1A, Supplementary Figure S1). During affinity maturation, B cells expressing these genes undergo rapid somatic evolution inside germinal centers, where activation-induced deaminase introduces point mutations at rates far above the germline substitution rate [8] and selection for antigen binding acts over days to weeks [9]. This accelerated regime generates densely sampled phylogenies of variants within a single host [10], allowing us to detect entrenchment by inferring selection on germline-encoded residues.

Prior work on entrenchment has focused predominantly on intra-protein epistasis as its source. Indeed, Starr et al. [3] found that intra-protein epistasis accounts for nearly all entrenchment in Hsp90, but they noted that the balance between intra- and inter-molecular contributions may differ across protein systems. Antibodies offer a distinctive system: during B cell development, a germline IGHV gene is joined to D and J gene segments by V(D)J recombination, the resulting heavy chain pairs with a light chain from a separate locus, and the assembled complex then binds antigen. During affinity maturation, selection acts in context of the full complex, so IGHV-encoded residues can experience epistasis from multiple sources: from the IGHV residues themselves, from the D and J gene segments recombined with the IGHV gene, and from the additional proteins the heavy chain interacts with: the light chain and the antigen. Notably, the D and J genes, the light chain, and the antigen are all genetically independent from the IGHV gene and evolve separately, so their constraints would not have been gradually acquired along the germline lineage. Here, we study entrenchment across IGHV genes, asking which germline amino acid differences are entrenched and whether their entrenchment arises from constraints within the IGHV or from interactions with these genetically uncoupled partners.

Beyond identifying the sources of epistasis, entrenchment analysis can reveal which germline differences are selectively maintained rather than neutral. IGHV germline diversity influences V-J pairing [11], heavy-light chain pairing [12, 13], and convergent antibody responses [14]. Certain germline-encoded motifs even bind specific amino acid residues in antigens, driving immunodominant public responses [15]. However, not all germline diversity is necessarily functional: some amino acid differences may be selectively neutral, having fixed through genetic drift.

We test for entrenchment using DASM (Deep Amino acid Selection Model), a deep-learning model trained to quantify selection on germline-encoded amino acids from antibody repertoire data [16]. The model infers selective pressures along phylogenetic trees of millions of affinity-matured antibody sequences. We examine entrenchment at two levels of sequence divergence: within V gene families, where most gene pairs differ at up to ~10% for IGHV4, ~15% for IGHV1, or ~20% for IGHV3, and between families, where they differ at 25–40% (Supplementary Figure S1). Within V gene families, we find that entrenched sites concentrate at the borders of the complementarity-determining regions (CDRs, the antigen-binding loops) and contact multiple genetically independent partners, and we characterize these interactions using structural and physicochemical analyses. Between V gene families, additional entrenched sites appear in framework regions (the scaffolding positions between CDRs), and we examine candidate structural explanations at selected sites. Validation using observed mutation frequencies in human repertoires corroborates these predictions at sites with sufficient data. Together, these results suggest that entrenchment in antibodies arises from both intra-heavy-chain structural constraints and epistasis with genetically uncoupled partners.

## Results

### Identifying entrenched germline amino acids within V families

Under entrenchment, reciprocal substitutions between two germline amino acids found at the same site (e.g., W→G and G→W) are strongly disfavored (Figure 1B). DASM selection factors quantify how selection shifts the probability of each substitution relative to neutral expectation: a log selection factor above zero indicates the substitution has a beneficial effect, a value of zero indicates a neutral effect, and a value below zero indicates a deleterious effect. We classify a site as entrenched for a given amino acid pair when both reciprocal substitutions have median natural-log selection factors below −1, i.e., occurring at less than 37% of the neutral rate (Figure 1). Because the strength of entrenchment is continuous, we chose this cutoff to focus on sites with the strongest predicted effects.

We began by examining entrenchment between V genes within the same V family, where most gene pairs differ at less than ~20% of their amino acids (Supplementary Figure S1). To detect entrenchment, we identified sites where different V genes have divergent germline amino acids and used DASM to compare predicted selection factors for reciprocal substitutions between these residues (Figure 1). We applied DASM to a held-out repertoire dataset, restricting to sequences at the most recent common ancestor of each clonal family, where training data are most abundant and selection factors are not yet shifted by somatic mutations accumulated during affinity maturation. We further restricted to single-nucleotide substitutions, as multi-nucleotide substitutions are rare in the training data and may yield less reliable selection-factor estimates.

For each site and pair of germline amino acids, we measured the median predicted selection factor for substitutions converting one amino acid toward the other. This approach aggregates across all V genes within a family that share the same amino acid at a given site, yielding more robust median log selection factor estimates. Per-V-gene-allele medians confirmed the consistency of this grouping (ICC > 0.96 for all families; Supplementary Figure S5, Methods).

### Entrenchment between V genes within the same V family clusters at CDR borders

**Figure 2:**
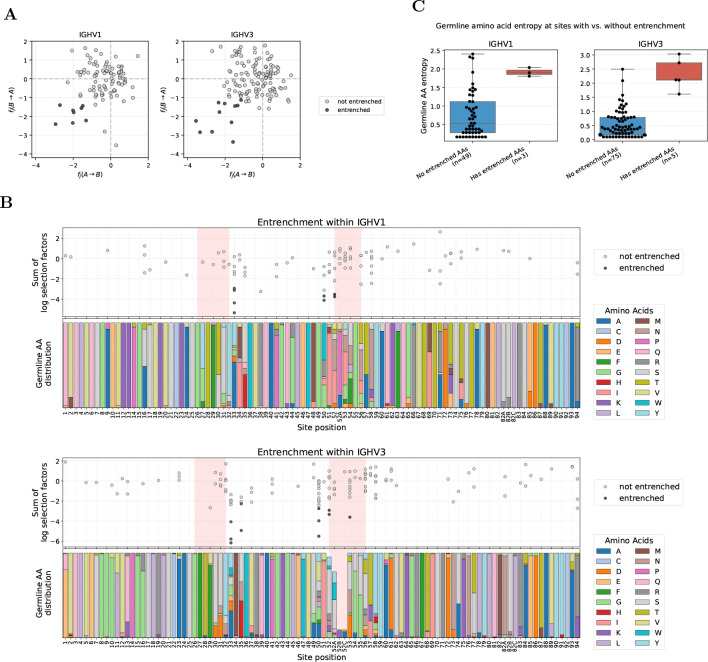
Entrenchment analysis within V families identifies sites at CDR borders with high germline diversity. **(A)** Reciprocal log selection factors between amino acids A and B, annotated as $f_{j}(A\to B)$ and $f_{j}(B\to A)$, for amino acid pairs at sites where V genes within a family differ in germline identity, shown for IGHV1 (left) and IGHV3 (right). Each dot represents one amino acid pair at one site; entrenched pairs (both reciprocal log selection factors < −1) are highlighted as black dots. **(B)** Entrenchment mapped along the V gene sequence (IGHV1 top, IGHV3 bottom). Top subpanels show the sum of reciprocal log selection factors ($f_{j}(A\to B)+f_{j}(B\to A)$) for each amino acid pair at each site, with entrenched pairs highlighted as in (A). Pink shading highlights CDR regions. Bottom subpanels show the distribution of germline amino acids across V genes in each family. Sites with germline variability but no points (e.g., site 2 in IGHV3) lack sufficient data after filtering for single-nucleotide substitutions and requiring ≥10 observations per amino acid (see Methods for full filtering criteria). Analogous analysis for IGHV4 is shown in Supplementary Figure S6. **(C)** Shannon entropy comparison at sites with versus without entrenched amino acid pairs. Sites with entrenched pairs show higher germline diversity, though there is overlap between distributions. Sites with only one germline amino acid across V genes in the family (entropy = 0) are excluded. A complete list of all entrenched pairs is provided in Supplementary Table S1. Site numbering follows the Chothia scheme [17] throughout.

We focused our within-family entrenchment analysis on IGHV1 and IGHV3, the two families with the greatest germline diversity and abundant repertoire data. Across all sites, we tested for entrenchment among 95 and 130 pairs of germline amino acids in IGHV1 and IGHV3, and found 8 and 11 entrenched, respectively (Figure 2A; Supplementary Figure S9). Most entrenched sites are retained under stricter thresholds down to −1.4 (Supplementary Figures S2, S3, and S4).

Sites showing entrenchment (hereafter, entrenched sites) cluster predominantly at CDR boundaries (Figure 2B). Specifically, these sites concentrate at the end of CDR1 and beginning of CDR2 in both V families, a pattern also observed in IGHV4 (Supplementary Figure S6). We also detect a single entrenched amino acid pair at CDR2 site 53 in IGHV3 (usually not adjacent to site 52 due to Chothia insertion codes such as 52A). These sites exhibit high germline diversity (Figure 2B, bottom panels). Because entrenchment requires at least two germline amino acids at a site, entrenched sites necessarily have nonzero diversity; however, high diversity alone does not predict entrenchment. The Shannon entropy distributions of entrenched and non-entrenched sites overlap, and several highly diverse sites show no evidence of entrenchment (e.g., sites 52A–54 in IGHV1 and 52A, 56–58 in IGHV3; Figure 2B).

### Entrenched sites contact multiple genetically independent partners

**Figure 3:**
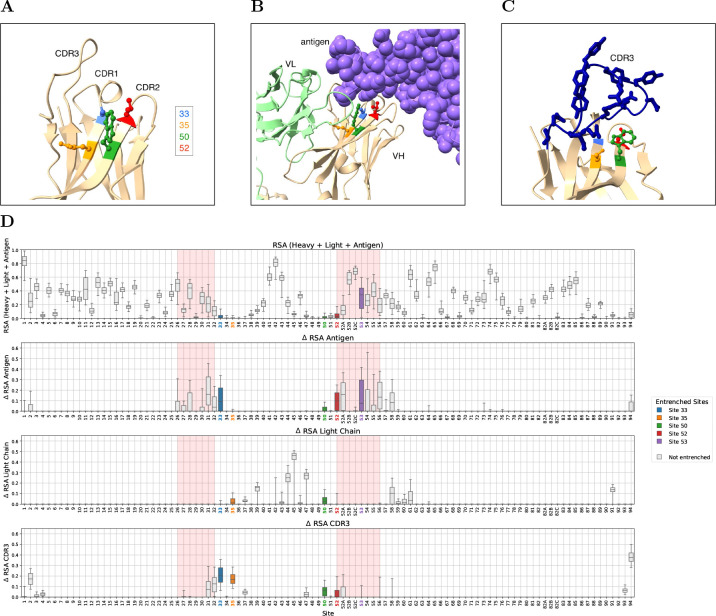
Within-family entrenched sites lie on CDR-scaffolding beta sheets and contact genetically independent partners. **(A)** Heavy chain of an example antibody (PDB: 7L7D, resolution 2.5Å), an IGHV1 antibody binding the SARS-CoV-2 spike protein, showing the spatial arrangement of entrenched sites 33, 35, 50 and 52 with annotated CDRs. These sites cluster at or near the edges of beta sheets that scaffold the CDR loops. **(B)** Same structure with light chain (green) and antigen (purple). Entrenched sites contact the antigen and light chain. **(C)** Heavy chain of a second IGHV1 antibody (PDB: 6B0G, resolution 1.9Å), an IGHV1–18 antibody binding Pfs25, showing CDR3 loop (blue) contact with entrenched sites. In this structure, these sites contact the CDR3 but not antigen or light chain, contrasting with (B). **(D)** RSA analysis for IGHV3 family at germline encoded sites with entrenched amino acids. Row 1: Relative solvent accessibility (RSA) in the full complex. Row 2: Change in RSA upon antigen removal (antigen effect). Row 3: Change in RSA upon light chain removal (light chain effect). Row 4: Change in RSA upon CDR3 removal (positions 95–102). The corresponding RSA analysis for IGHV1 family is shown in Figure S7.

We next asked what molecular interactions might cause the entrenchment. When we mapped the entrenched sites onto heavy-chain crystal structures, we found a striking pattern: four of the five sites (33, 35, 50, and 52) cluster closely together on two strands of a beta sheet at the base of the CDR1 and CDR2 loops, right at the interface of the heavy-light-antigen complex (Figure 3A–C). These sites often contact three regions whose sequences are genetically independent of the IGHV germline: the light chain (encoded at a separate locus), the antigen, and the CDR-H3 loop (encoded by D and J gene segments joined by V(D)J recombination with additional junctional diversity).

To systematically quantify these interactions, we examined antibody crystal structures from the SAbDab database that include a heavy chain, light chain, and antigen [18] (510 structures for IGHV1 and 846 for IGHV3; see Methods for filtering criteria). For each structure, we computed the relative solvent accessibility (RSA) of each heavy-chain residue in the full complex, as well as the change in RSA (ΔRSA) upon removing the antigen, the light chain, or the heavy chain CDR3 (here defined as positions 95–102, extending one position beyond the Chothia CDR-H3 boundaries on each side to capture the variable part of the V(D)J junction). Unlike the CDR3 loop, the adjacent FR4 is a conserved framework segment that presents consistent selection pressure across antibodies, so it was retained despite also being J-gene-encoded. The four clustered entrenched sites (33, 35, 50, 52) are highly buried in the full complex, while site 53 is more solvent exposed (Figure 3D, top row). All three genetically independent partners contribute to burial at these sites, with the relative importance varying by position: sites 33 and 52 are buried primarily by antigen and the CDR3 loop, site 35 by the CDR3 loop and light chain, site 50 roughly equally by all three, and site 53 primarily by antigen (Figure 3D, rows 2–4).

**Figure 4:**
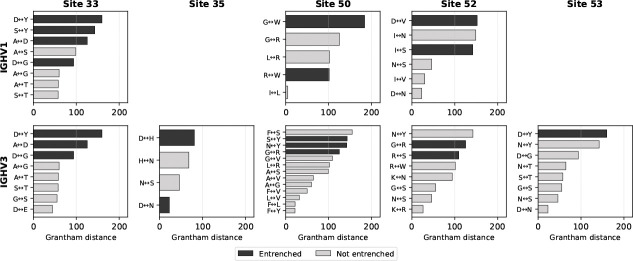
Entrenched amino acid pairs are more physicochemically distinct than non-entrenched pairs. Grantham distance for each amino acid pair at the five within-family entrenched sites, shown separately for IGHV1 (top row) and IGHV3 (bottom row). Sites 35 and 53 appear only in IGHV3, as they are not entrenched within IGHV1. Pairs are sorted by Grantham distance; black bars indicate entrenched pairs and grey bars indicate non-entrenched pairs. Entrenched pairs tend to have larger Grantham distances, indicating greater physicochemical difference between the two amino acids. A stratified permutation test across 8 V-family × site groups confirms this trend (*p* < 0.0001; 10,000 permutations).

To further characterize these interactions, we examined both the physicochemical properties and the structural contacts of amino acid pairs at entrenched sites. Entrenched amino acid substitutions involve more physicochemically distinct amino acids than non-entrenched substitutions at the same sites, as measured by Grantham distance (stratified permutation test across 8 (V-family × site) groups, *p* < 0.0001; Figure 4). Moreover, different germline amino acids at the same site establish distinct contact patterns with antigen, light chain, and CDR-H3 (Supplementary Figure S8), though some variability exists among structures with the same amino acid. At site 33, where amino acid pairs are highly repeated between IGHV1 and IGHV3, most of the same amino acid pairs are entrenched in both families (e.g., aspartic acid (D)↔glycine (G) and aspartic acid (D)↔tyrosine (Y)), while other, more physicochemically similar pairs are tolerable in both (e.g., alanine (A)↔glycine (G) and serine (S)↔threonine (T)), corroborating the Grantham distance analysis (Figure 4).

Together, these results suggest that entrenchment of V-gene residues could arise through epistatic interactions between the V gene and each of these binding partners. The high within-family germline diversity at these sites indicates that the constraints are not imposed by the heavy chain fold alone, since closely related V genes tolerate diverse amino acids at these positions. However, not all sites with partner contact are entrenched: sites 31 and 32 have both antigen and CDR3 loop contact without being entrenched, and multiple other sites have contact with one partner without being entrenched (Figure 3D), suggesting that partner contact alone is not sufficient.

### Entrenchment between V families suggests intra-protein structural constraints

We next examined entrenchment between more distantly related proteins by comparing V genes from different families. V genes from different families typically differ in approximately 25–40% of their amino acids (Supplementary Figure S1), representing evolutionary divergence that occurred over a longer timescale than within-family variation. We applied a similar approach to identify entrenched sites between V families, focusing on sites with germline divergence and assessing reciprocal purifying selection patterns. Again, groups of V genes within a V family that share the same amino acid at a site were aggregated to obtain more robust median log selection factor estimates for that site.

**Figure 5:**
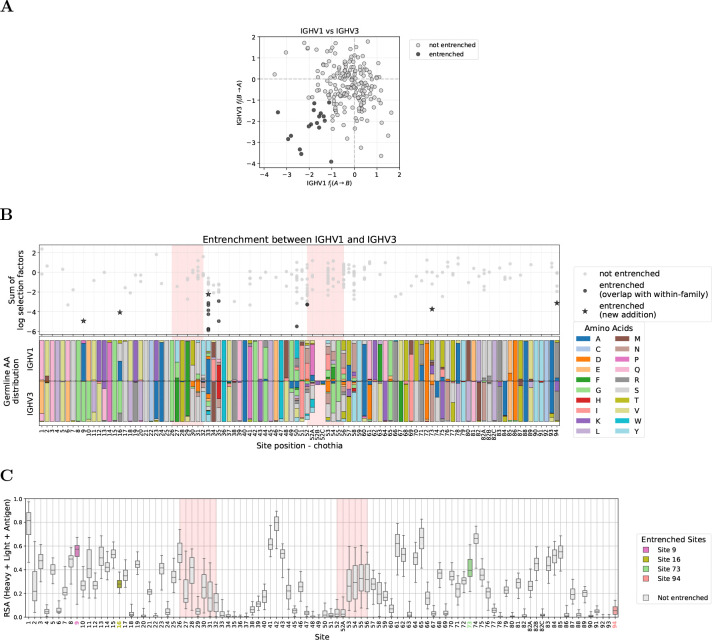
Entrenchment between more distantly related homologs retains within-family sites and adds framework positions. **(A)** Reciprocal log selection factors ($f_{j}(A\to B)$) vs. $f_{j}(B\to A)$) for amino acid pairs at sites where IGHV1 and IGHV3 differ in germline identity; entrenched pairs (both < −1) are highlighted. **(B)** Entrenchment between IGHV1 and IGHV3 mapped along the V gene sequence. Top panel shows the sum of reciprocal log selection factors for each amino acid pair at each site. Light grey dots indicate non-entrenched pairs; dark dots indicate entrenched pairs that overlap with within-family entrenched sites; stars indicate entrenched pairs not entrenched within either family (new additions). Pink shading highlights CDR regions. Bottom panel shows the distribution of germline amino acids at each site in IGHV1 (above axis) and IGHV3 (below axis). Analogous analysis for IGHV3 vs IGHV4 and IGHV1 vs IGHV4 is shown in Supplementary Figure S12. **(C)** Relative solvent accessibility (RSA) in the full antibody-antigen complex for IGHV1, highlighting sites entrenched only in the between-family analysis (i.e., not entrenched within either family). Extended RSA analysis including antigen, light chain, and CDR3 effects for both IGHV1 and IGHV3 is shown in Figure S10. These between-family-only sites show lower germline diversity than within-family entrenched sites (Supplementary Figure S11).

The between-family analysis predicted entrenchment at several sites, including four of the five sites predicted to be entrenched in the within-family analysis (Figure 5). Sites that were only predicted to be entrenched in the between-family analysis occur predominantly in framework regions and span a range of solvent accessibility values, from buried to highly exposed (Figure 5C, Figure S10). These sites show less within-family germline diversity than within-family entrenched sites (Supplementary Figure S11). Several, such as sites 9 and 73, are among the most solvent-exposed positions in the heavy chain. Most of these sites do not make contact with antigen, light chain, or CDR3, though CDR3-bordering site 94 does contact both antigen and the CDR3 loop (Figure S10).

For the majority of between-family-only entrenched sites, the lack of contact with genetically independent partners suggests that their entrenchment arises from structural constraints intrinsic to the part of the heavy chain encoded by the V gene rather than from epistasis with genetically uncoupled partners.

**Figure 6:**
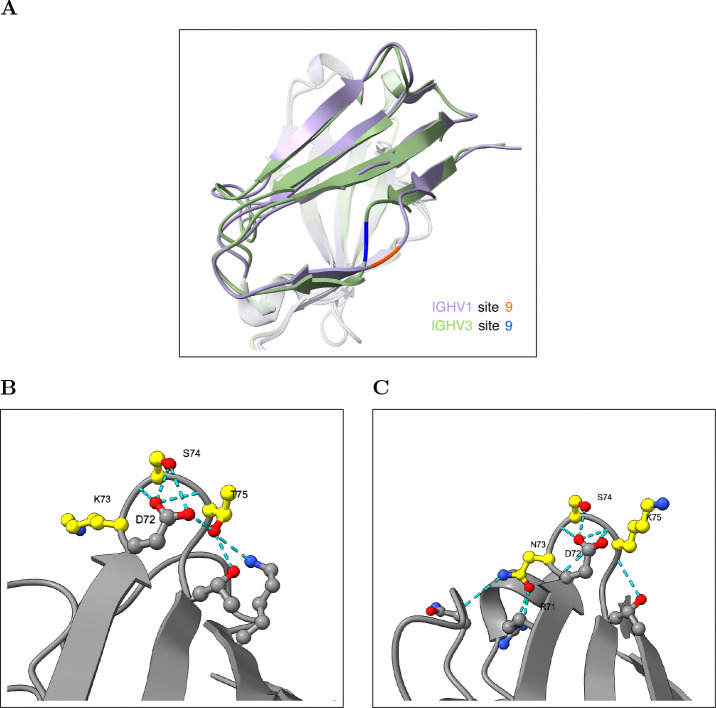
Entrenched sites 9 and 73 are in regions with structural differences between IGHV1 and IGHV3. **(A)** Structural alignment of IGHV1 (purple; PDB: 3SQO, 2.7 Å resolution) and IGHV3 (green; PDB: 1NL0, 2.2 Å resolution) near site 9, where IGHV1 carries Alanine (orange) and IGHV3 carries Glycine (blue). The two families adopt divergent backbone conformations at sites 7–10. Backbone dihedral angle analysis across SAbDab structures is shown in Supplementary Figure S13. **(B)** IGHV1 hydrogen bonding network (PDB: 8G3Z): K73 lacks consistent framework hydrogen bond partners, while T75 anchors inward via hydrogen bonds. Sites 73–75 are highlighted in yellow and hydrogen bonds are shown as cyan dashes. **(C)** IGHV3 hydrogen bonding network (PDB: 4H8W): N73 is anchored by hydrogen bonds to R71 and a CDR-H2 backbone carbonyl; colored as in (B). H-bond analysis across all eight examined structures is reported in Supplementary Table S2.

We investigated several of these entrenched positions in detail and found that they lie in regions with structural differences between IGHV1 and IGHV3; however, it is not immediately obvious how these differences give rise to entrenchment. For example, site 9 has an entrenched alanine/glycine polymorphism between IGHV1 and IGHV3 and lies in a region spanning sites 7–10 where the two families adopt distinct backbone conformations, in contrast to the generally conserved backbone geometry in most of the remaining structure (Figure 6A; Supplementary Figure S13). While glycine at site 9 in IGHV3 assumes a wider range of backbone geometries than alanine at the same site in IGHV1, these distributions largely overlap, making it difficult to explain entrenchment at this site through backbone geometry alone. Site 73 has an asparagine/lysine polymorphism between IGHV3 and IGHV1 and lies in a loop spanning sites 72–75. In IGHV3, asparagine at site 73 is consistently anchored by hydrogen bonds to arginine at site 71 and a CDR-H2 backbone carbonyl across all examined structures, while lysine at site 73 in IGHV1 forms no framework hydrogen bonds and only inconsistent contact with CDR-H2 (Figure 6B–C, Supplementary Table S2). This provides a partial structural explanation: replacing asparagine with lysine in IGHV3 would disrupt these stabilizing contacts, though the structural basis for why the reverse substitution would be disfavored in IGHV1 is less clear.

### Validation of DASM predictions using mutation rates

DASM is a transformer-encoder model that quantifies how selection shifts the probability of each substitution relative to neutral expectation [16]. Neutral expectations are provided by Thrifty, a kmer-based model that estimates the expected mutation probability of each substitution from its local sequence context [19]. To validate these predictions, we independently estimated selection using a simplified approach based on mutation counts.

To do so, we calculated the ratio of observed to expected mutation rates for each site and substitution by comparing mutation counts in productive sequences, which are subject to selection, to counts in out-of-frame (non-productive) sequences, which evolve neutrally with respect to amino acid changes [20, 19], accounting for phylogenetic branch lengths. We used a held-out test dataset for the productive mutation counts and a separate dataset that has not undergone selection for the out-of-frame counts; the latter was not used to train the neutral mutation model, though it was used to fit the multi-hit correction (3 parameters only; see Methods). To ensure stable rate ratio estimates, we required ≥ 5 mutation counts in the out-of-frame data, retaining 1180 out of 3218 substitutions.

**Figure 7:**
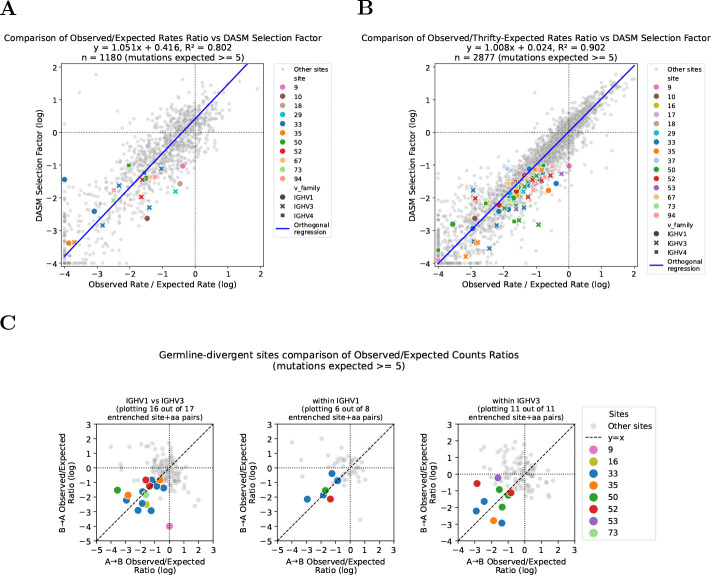
Observed mutation rate ratios support DASM entrenchment predictions where data are sufficient. **(A)** Validation using out-of-frame sequences as neutral baseline. The log ratio of observed to expected mutation rates correlates with log selection factors predicted by our model for sites and substitutions with sufficient mutation counts (expected counts ≥ 5). Each point represents one V-family, site, parent amino acid, and target amino acid combination. Correlation is calculated for all points; entrenched sites and substitutions that pass filtering are highlighted in color. **(B)** Validation using Thrifty-inferred expected mutation rates. The log ratio of observed mutations (from productive sequences) to expected mutations (inferred from Thrifty neutral model) correlates with log selection factors predicted by DASM. This approach allows validation of more sites compared to the out-of-frame-based approach, while maintaining strong correlation with DASM predictions. Entrenched sites and substitutions that pass filtering are highlighted in color. **(C)** Pairwise validation of entrenched substitutions. Log rate ratios in both directions (A→B and B→A) as calculated in (B) for all possible pairs of reciprocal substitutions, while highlighting those identified as entrenched by DASM. Not all entrenched substitutions that appear in (B) appear here, as some do not have sufficient data for both directions of the pair to be included in this plot. Analogous validation for comparisons involving IGHV4 is shown in Supplementary Figure S14.

For substitutions passing this filter, the log ratio of observed to expected mutation rates correlated with DASM’s predicted log selection factors (Figure 7A), indicating that the model captures genuine selective constraints. This approach could directly validate only 21 of 83 substitutions involved in entrenchment, as many had too few mutations in the out-of-frame sequences for stable rate ratio estimates. Of those 21, all showed purifying selection and 18 (86%) fell below the −1 entrenchment threshold, including substitutions at each of the four CDR-bordering sites (33, 35, 50, and 52). Non-productive sequences are far fewer than productive ones, making the out-of-frame data the limiting factor in this analysis.

We thus used a complementary approach: instead of out-of-frame data as neutral baseline, we inferred expected counts directly from Thrifty and used the much larger training dataset to increase statistical power (see Methods). Because Thrifty estimates neutral rates from local sequence context rather than per-site counts, it should provide more stable expected rates at sites where out-of-frame data are too sparse for reliable empirical estimates, though confirming this directly would require larger out-of-frame datasets. This retained 2877 out of 3247 substitutions after applying the same filter.

Using this approach, the correlation between DASM selection factors and observed-to-expected mutation rate ratios remained strong (Figure 7B). To validate predicted entrenchment for specific substitution pairs, we examined whether both directions of each reciprocal pair show purifying selection (Figure 7C). Of 36 entrenched reciprocal pairs, 33 had sufficient data in both directions; of these, 29 (88%) showed log rate ratios below −0.5 and 19 (58%) below −1 in both directions, covering all four CDR-bordering sites. The sole exception to purifying selection was IGHV1 site 9 A→G, which had a log rate ratio near zero. This discrepancy arises because the gene IGHV1–18 has atypically positive selection at this site (Figure S5) and contributes disproportionately to the expected counts due to higher neutral mutability, so it dominates the aggregate observed-to-expected ratio despite most V genes in this family showing strong purifying selection. The median-based aggregation across V genes we use for DASM is robust to such outliers.

All entrenched amino acid substitutions from both within- and between-family comparisons are listed in Supplementary Table S1, and their DASM-based selection factors and rate ratios from both validation approaches are summarized in Supplementary Table S3.

## Discussion

Previous studies have attributed entrenchment to intra-protein epistasis accumulating over deep evolutionary time [2, 3, 1, 4]. Our results suggest that epistasis from genetically uncoupled partners can also drive entrenchment: within V gene families, entrenched sites contact antigen, light chain, and the CDR-H3 loop — all encoded independently of the IGHV germline — even though the same sites tolerate diverse amino acids across the germline repertoire.

The relative contribution of genetically uncoupled and intra-IGHV epistasis shifts with evolutionary distance. Among closely related V genes, intra-chain structural constraints appear to be largely shared; entrenched sites instead cluster at CDR borders, where interactions with antigens, light chains, and CDR-H3 loops could lock in germline amino acids during affinity maturation. At deeper divergence between V gene families, additional entrenched positions appear, which seem likely to be due to intrinsic structural requirements of the heavy chain region encoded by the V gene, while the CDR border sites identified within families largely persist (Figure 5). In both cases, the constraints predate affinity maturation: V(D)J recombination, heavy chain folding, light chain pairing, and antigen binding all occur beforehand, so affinity maturation reveals rather than creates these constraints.

Our analysis aggregates selection signals across thousands of clonal families with diverse CDR-H3, light chain, and antigen combinations, averaging out partner-specific constraints. The entrenchment that persists reflects general constraints on IGHV-encoded residues that hold across the combinatorial diversity of partners encountered during affinity maturation. Different V genes carry different amino acids at these same positions, each favored by selection in its own sequence context, suggesting that the entrenched residues help define each V gene’s distinct mode of engaging its partners.

Within-family entrenchment concentrates at CDR1 and CDR2 edges rather than in CDR centers. CDR-central sites tend to be more solvent-exposed, even in the full antibody-antigen complex, and among the genetically independent partners, they primarily contact only the antigen rather than the light chain or CDR-H3 loop. Additionally, many CDR-central sites experience diversifying selection during affinity maturation [21], which works against the reciprocal purifying selection that defines entrenchment. CDR border sites, by contrast, occupy multiple roles: they facilitate contact with the CDR-H3 loop, light chain, and antigen, and they are also part of the framework that scaffolds CDR1 and CDR2. These multiple structural and functional roles might make entrenchment more common at these sites. Entrenched sites partially overlap with germline-encoded antigen-binding (GRAB) motifs [15] at sites 33, 52, and 94, but the two approaches capture different signals: GRAB motifs identify residues that contact specific antigen amino acids, whereas entrenchment detects any site under reciprocal purifying selection, regardless of the mechanism. Conversely, some GRAB sites may not appear entrenched because DASM averages selection across thousands of clonal families with diverse antigens, which could mask antigen-specific constraints.

The antibody system combines properties rarely available together: dozens of homologous germline genes, combinatorial assembly by V(D)J recombination and chain pairing, diverse antigens, and fast somatic evolution [10]. This architecture offers a unique opportunity to observe multiple levels of epistatic constraints: within a single germline-encoded segment (intra-IGHV constraints), between independently recombined segments of the same polypeptide (IGHV versus CDR3), and between separate proteins that function as a complex (heavy chain versus light chain and antigen). Because each B cell assembles a new combination of these before affinity maturation, the system is a natural experiment for detecting epistasis from genetically uncoupled regions.

MHC molecules are a particularly relevant biological parallel: polymorphic alleles at each locus bind diverse peptide repertoires, creating conditions where background-dependent constraints and entrenchment-like patterns are expected. Balancing selection has been established at MHC loci using population-level analysis of germline alleles [22, 23]; performing analogous germline-level analyses for IGHV is more difficult because the immunoglobulin locus is challenging to assemble due to extensive segmental duplication and repetitive sequence [24, 25]. However, the somatic evolution of antibodies provides a complementary window into these dynamics by allowing us to observe selection on germline-encoded amino acids across a dense phylogeny of variants within individuals. Our findings also raise the possibility that germline diversity at entrenched sites is itself maintained by balancing selection: if different V genes access distinct antigen-binding solutions through the amino acids at these positions, selection could favor retaining multiple V genes rather than permitting fixation of any single variant.

Three methodological limitations should be noted. First, we restricted our analysis to single-nucleotide substitutions, so not all potentially entrenched substitutions can be assessed. Second, affinity maturation could itself generate new entrenchment as compensatory somatic mutations accumulate among somatically acquired residues, a possibility our germline-focused framework does not address. Third, not all DASM predictions can be independently validated with counts, as both the out-of-frame and Thrifty-based approaches retain only a subset of testable substitutions. Where both are available, the strong correlation between DASM and counts-based estimates supports the model’s ability to generalize. Model-based inference can extend predictions to data-sparse sites where counts-based approaches lack statistical power, but confirming whether these predictions reflect genuine biological constraints will require larger repertoire datasets or experimental validation.

The patterns we observe are consistent with epistasis from genetically uncoupled partners as a source of within-family entrenchment, and recent experimental work offers independent support. Phillips et al. [26] found epistasis between heavy and light chain mutations in the CH65 broadly neutralizing anti-influenza antibody at heavy chain sites 33, 35, and 52, matching three of the four positions we identify as entrenched within V families. Their combinatorial library tested somatic mutations in a single antibody lineage. Extending deep mutational scanning [27] to germline substitutions across diverse light chain and CDR-H3 contexts would more directly test whether genetically uncoupled epistasis constrains the germline differences we observe.

More broadly, our findings illustrate that constraints from genetically independent partners — whether inter-molecular or inter-segmental — can shape germline-encoded residues in ways that are difficult to detect when proteins are analyzed in isolation. Somatic evolution within the antibody repertoire provides a rare opportunity to observe these constraints directly, and similar approaches may extend to other systems where related proteins evolve together with diverse partners.

## Materials and methods

### Data, code, and models

We used four human heavy chain antibody repertoire datasets (Table 1), previously processed by the DASM (productive data) and Thrifty (out-of-frame data) publications [16, 19]. Sequences from each dataset are partitioned into clonal families with inferred phylogenetic trees. A sequence is considered out-of-frame if either of the conserved codons for cysteine or tryptophan bounding the CDR3 is out-of-frame with the germline V gene.

The preprocessed Rodriguez, Jaffe, and Tang datasets are available as per these previous papers at Zenodo (https://zenodo.org/records/15931791) and the Tang-SHM dataset at Dryad (https://datadryad.org/dataset/doi:10.5061/dryad.np5hqc044).

The DASM model used for “entrenchment detection using DASM selection factors”, dasm 4m-v1jaffeCC+v1tangCC-joint, was trained as described in [16] on JaffeCC and TangCC heavy chain datasets. The PCP counts for Jaffe and Tang in Table 1, which are used for validation (Approach 2) of selection factors, reflect slightly different filtering steps than for the datasets used in the DASM training (e.g., the validation data removes all sequences containing ambiguous nucleotides, while the training pipeline instead filters for conserved cysteines and removes parent–child pairs whose mutations occur only in ambiguous codons), as described in [16]. The neutral model used for “Validation of DASM inferences using mutation rate ratios”, ThriftyHumV0.2–59, was trained as described in[19].

Analysis code for this study will be made available upon journal submission.

### Entrenchment detection using DASM selection factors

To identify entrenchment, we used DASM, a deep amino acid natural selection model [16], to predict selection factors on a dataset not used to train the model (Rodriguez). We used an unseen dataset to ensure that the selection factors represent generalizable predictions rather than overfitting artifacts that could produce spurious entrenchment signals. Because DASM predicts selection factors per sequence using context learned from its ~880,000-PCP training set (JaffeCC and TangCC combined), the Rodriguez test set supports well-resolved per-site estimates despite being smaller than the training data. We filtered sequences, sites, and selection factors so that:

- The parent sequence is the most recent common ancestor of each clonal family (i.e., root of the phylogenetic tree; parent–child pair depth = 2), yielding 12,944 PCPs for the Rodriguez dataset.
- Only sites where the parent amino acid is encoded by a germline codon are used.
- Target amino acid can be reached from parent sequence codon with a single-nucleotide substitution.

We inferred the median selection factor per combination of site, parent amino acid, target amino acid, and V family, and required at least 10 observations per combination.

For each site j, we considered reciprocal amino acid pairs (A,B) where both A→B and B→A substitutions have an available median selection factor after the above steps. Let:

(1) $$f_{j}(A\to B)=\text{median}\;\{log\;\text{selection}\;\text{factor}\;\text{for}\;A\to B\;\text{at}\;\text{site}\;j\}$$

(2) $$f_{j}(B\to A)=\text{median}\;\{log\;\text{selection}\;\text{factor}\;\text{for}\;B\to A\;\text{at}\;\text{site}\;j\}.$$

A site is classified as **entrenched** for the amino acid pair (A,B) if:

(3) $$f_{j}(A\to B)<-1\;\text{and}\;\;f_{j}(B\to A)<-1$$

Comparison can be made between different germline amino acids within a V family or between different V families. This criterion requires both reciprocal substitutions to be less than 37% as likely as under neutral selection, indicating strong bidirectional purifying selection.

### Validation of within-family grouping

Our entrenchment analysis aggregates selection factors across V genes within a family that share the same germline amino acid at a given site. Because these V genes differ at up to ~20% of other positions, this pooling assumes that selection landscapes at a given site are similar across V genes in the group. To test this assumption, we computed per-V-gene median log selection factors using the same filtering criteria as the family-level analysis. For each combination of site, parent amino acid, target amino acid, and individual V gene, we required ≥3 observations. We assessed agreement using two metrics. First, the intraclass correlation coefficient (ICC), computed with a one-way random effects model partitioning variance into between-group (substitution identity) and within-group (V gene identity) components, was 0.97, 0.96, and 0.98 for IGHV1, IGHV3, and IGHV4 respectively. Second, threshold agreement (the fraction of individual V gene medians at family-level entrenched sites that independently fall below the −1 threshold) was 94%, 93%, and 92% for the three families. In the few cases of disagreement, dissenting V gene medians generally remained negative, indicating weaker purifying selection rather than a qualitative reversal, with the exceptions of site 9 in IGHV1 where two alleles of the same V gene (IGHV1–18) out of 21 showed positive medians and site 94 in IGHV3 where some V genes showed medians near zero (Supplementary Figure S5). This validation applies equally to the between-family analysis, which relies on the same within-family aggregation.

### Solvent accessibility and backbone angle analysis

To characterize the structural context of entrenched sites, we analyzed antibody-antigen crystal structures from the Structural Antibody Database (SAbDab) [18]. Structures were filtered to include only human antibodies with complete heavy chain, light chain, and antigen chains present. Structures with multiple antibodies were retained when chain sequences were consistent across all entries for each PDB. We assigned V genes using BLAST and identified unmutated positions by comparison to germline references from the AIRR Community OGRDB (https://ogrdb.airr-community.org/germline_set/75).

We calculated solvent accessible surface area (SASA) using the DSSP algorithm [31] with Wilke reference values [32] to obtain relative solvent accessibility (RSA) on a 0–1 scale. To isolate the contributions of different binding partners to heavy chain accessibility, we computed SASA in four scenarios: (1) the complete antibody-antigen complex (heavy + light + antigen chains), (2) the antibody alone (heavy + light chains), (3) the heavy chain in isolation, and (4) the heavy chain with the CDR3 loop (positions 95–102) removed. The antigen effect was calculated as the difference in RSA between the complete complex (scenario 1) and the antibody alone (scenario 2). The light chain effect was calculated as the difference in RSA between the antibody alone (scenario 2) and the heavy chain in isolation (scenario 3). The CDR3 effect was calculated as the difference in RSA between the heavy chain in isolation (scenario 3) and the heavy chain with the CDR3 loop removed (scenario 4). The adjacent FR4 (positions 103–113), though also encoded by the J gene segment, was retained in scenario 4 because the goal is to isolate the effect of the somatically diversified CDR-H3 loop; FR4 is a conserved framework segment, not part of this variable region. Backbone dihedral angles (*ϕ* and *ψ*) were calculated using BioPython’s PPBuilder [33] to characterize local backbone geometry at each residue. All residue positions were numbered using the Chothia scheme [17]. The SAbDab database contains a biased sample of binding contexts with a large focus on therapeutically relevant antibodies, so the structural patterns observed may not be fully representative of the broader antibody repertoire.

### Grantham distance analysis

Grantham distance is a measure of physicochemical dissimilarity between amino acids based on composition, polarity, and molecular volume [34]. To test whether entrenched substitutions involve greater physicochemical change, we compared Grantham distances between entrenched and non-entrenched amino acid pairs. Because different sites have distinct amino acid repertoires, we used a stratified permutation test: within each V-family × site combination, we permuted the entrenched/non-entrenched labels (10,000 permutations), computed the per-stratum mean Grantham distance difference (entrenched minus non-entrenched), and averaged across strata. The one-sided *p*-value is the proportion of permuted statistics greater than or equal to the observed value.

### Structural analysis of FR3 hydrogen bonding networks

To investigate the structural basis of the observed entrenchment at site 73, we analyzed structures of Fab fragments from the Protein Data Bank (PDB). Structures were selected based on the following criteria: (1) human antibodies, (2) heavy chain V gene assigned to either IGHV1 or IGHV3 family, (3) unmutated germline residues at positions 71–75, (4) presence of the entrenched amino acids at position 73, and (5) resolution ≤3.0 Å. V gene assignments were performed using partis [35], and unmutated positions were identified by comparison to germline references from the AIRR Community OGRDB (https://ogrdb.airr-community.org/germline_set/75). Four structures meeting these criteria were analyzed for each family: IGHV1 (PDB: 7X29, 2NY6, 6VY4, 8G3Z; resolution 2.0–2.8 Å) and IGHV3 (PDB: 4H8W, 3BN9, 6ULE, 6PPG; resolution 1.85–2.75 Å). For each structure, PDB residue numbers were mapped to the Chothia numbering scheme by locating the conserved sequence motif at positions 71–75 (ADKST for IGHV1; RDNSK for IGHV3); mapping was verified individually per structure because PDB-to-Chothia offsets varied (direct correspondence in 6ULE, 4H8W, and 3BN9; +1 in 8G3Z, 7X29, 6VY4, and 6PPG; +3001 in 2NY6).

Structural analysis was performed using UCSF ChimeraX version 1.11.1 [36]. Because all heavy chain copies within each analyzed structure have identical sequence, we analyzed a single heavy chain per structure to avoid redundancy. Hydrogen bonds involving sites 73–75 were identified using the hbonds command with the restrict any option, which reports every H-bond in which the selected residues participate as either donor or acceptor against any partner (framework, CDRs, antigen). Default geometric criteria (0.4 Å and 20° tolerances relaxed from idealized per-atom-type geometry) give an effective absolute donor–acceptor distance cutoff of ~3.5 Å for N/O pairs. Each H-bond was classified by donor and acceptor type: backbone atoms are the amide N and carbonyl O, and all other donor/acceptor atoms are treated as sidechain.

### Validation of DASM inferences using mutation rate ratios

To validate entrenchment predictions from DASM and its underlying neutral model Thrifty, we compared selection factors against mutation rate ratios calculated from observed versus expected mutation frequencies. Under the DASM framework, the selection factor $f_{j}(X\to Y)$ quantifies how selection shifts the probability of substitution X→Y at site j relative to neutral expectation. When per-site mutation probabilities are small, substitution probabilities are approximately proportional to rates. The ratio of observed to expected mutation rates should therefore be approximately:

(4) $$\frac{{Rate}_{\text{obs},j}(X\;\to \;Y)}{{Rate}_{\text{expected},j}(X\;\to \;Y)}\approx f_{j}(X\;\to \;Y)$$

To estimate this ratio independently, we exploited the fact that productive sequences evolve under selection during affinity maturation, while sequences containing out-of-frame V(D)J junctions (also termed non-productive sequences) evolve neutrally with respect to amino acid changes and provide an estimate of the underlying mutational process [20, 19]. We used two complementary approaches to estimate expected mutation rates: one based on out-of-frame sequence counts and one based on predictions from the Thrifty neutral model.

### Mutation counts and rates

For both productive and out-of-frame datasets, we calculated mutation counts and rates from parent–child pairs (PCPs). We grouped all PCPs from the same V family, removing PCPs with ambiguous nucleotides (N) in their sequences. For productive sequences, which have undergone selection, we excluded leaf nodes to focus on mutations that have undergone subsequent rounds of selection [37]; for out-of-frame sequences, which evolve neutrally, we included all PCPs to maximize data. For each V family, site s, germline amino acid X, and target amino acid Y, let Count(X→Y∣s,Vfamily) denote the number of observed X→Y substitutions across all PCPs where the parent sequence retains the germline amino acid X at site s.

To calculate rates from counts, we accounted for the different branch lengths of different PCPs (defined separately for each approach below):

(5) $$Rate(X\;\to \;Y\;|\;s,\;V\;\text{family})=\frac{Count(X\;\to \;Y\;|\;X\;\text{is}\;\text{germline}\;\text{at}\;\text{site}\;s)}{\sum_{i\in {ℐ}_{X,s}}{\text{Branch}\;\text{length}}_{i}}$$

where ${ℐ}_{X,s}$ denotes the set of all PCPs in the V family where the parent sequence has germline amino acid X at site s.

### Approach 1: Out-of-frame data as neutral baseline

We calculated mutation rates separately for productive sequences (Rodriguez test dataset) and out-of-frame sequences (Tang-SHM dataset; Table 1). Although tang-shm is a held-out test dataset for Thrifty, Thrifty’s multi-hit correction component was trained using the tang-shm out-of-frame dataset, so Thrifty is not completely independent of the out-of-frame data. However, this multi-hit correction estimates only 3 parameters from the out-of-frame data, so the potential for data leakage affecting the validation is minimal. Out-of-frame sequences contain frameshifts in the VDJ recombination region but remain in-frame within the V gene region, allowing Chothia numbering and germline annotation and thus ensuring compatibility across datasets.

To calculate productive and out-of-frame mutation rates, we calculated branch lengths for every PCP as the frequency of synonymous nucleotide mutations:

(6) $${\text{Branch}\;\text{length}}_{i}=\frac{{\text{Number}\;\text{of}\;\text{synonymous}\;\text{mutations}}_{i}}{{\text{Sequence}\;\text{length}}_{i}}$$

We chose synonymous mutation-based branch lengths because synonymous mutations are under more comparable selective pressure in both productive and out-of-frame datasets than nonsynonymous mutations, which experience differential selection. Although synonymous mutations are not a perfectly clean neutral proxy [19], any resulting bias approximately cancels because synonymous-based branch lengths are used symmetrically in the productive and out-of-frame rate calculations. We then calculated the mutation rate for each amino acid substitution using (5).

The rate ratio for each V family, site, and amino acid substitution is:

(7) $$\text{Rate}\;ratio\left(X\to Y|s,\;V\;\text{family}\right)=\frac{{Rate}_{\text{observed}}(X\;\to \;Y\;|\;s,\;V\;\text{family})}{{Rate}_{\text{expected}}(X\;\to \;Y\;|\;s,\;V\;\text{family})}$$

To stabilize estimates and avoid artifacts from sparse data, we applied pseudocount smoothing (adding 0.5 to mutation counts), and retained only substitutions with at least 5 mutations observed in the out-of-frame dataset. This filtering retained 1,180 out of 3,218 testable substitutions for validation.

### Approach 2: Thrifty model as neutral baseline

As the size of the out-of-frame dataset limits validation coverage, we used a complementary approach that treats Thrifty’s neutral mutation model as ground truth to estimate expected counts. We used the much larger DASM model’s training dataset (Jaffe and Tang, Table 1) for this approach to increase statistical power. This dataset was used to train DASM, so this validation tests the DASM model but does not test whether its predictions generalize to unseen data.

To calculate Thrifty-predicted expected counts, we:

1. Calculated branch lengths as the total nucleotide mutation frequency multiplied by a scaling factor α=1.6:
(8) $$t_{i}=\frac{{\text{Number}\;\text{of}\;\text{nucleotide}\;\text{mutations}}_{i}}{{\text{Sequence}\;\text{length}}_{i}}\cdot \alpha$$
  where α is derived from regressing observed mutation frequencies against DASM-optimized branch lengths during DASM training. This correction accounts for purifying selection, which removes some expected mutations (1*/*1.6 ≈ 0.63, meaning observed mutations are approximately 63% as likely as neutral expectation). Because α is a single global scalar, it adjusts the overall scale of expected counts but cannot introduce a correlation where there is not one.
2. Converted Thrifty’s neutral mutation rates $\lambda_{i}^{\left(s\right)}$ at each site s for each PCP i into mutation probabilities: $p_{\text{mut},i}^{\left(s\right)}=1-e^{-\lambda_{i}^{\left(s\right)}\cdot t_{i}}$.
3. Multiplied by Thrifty’s conditional substitution probabilities $P_{\text{Thrifty}}(Y|X,s,i)$ and summed across the same PCPs used to count observed mutations:
(9) $${Count}_{\text{expected}}(X\to Y|s,\;V\;\text{family})=\sum_{i\in {ℐ}_{X,s}}p_{mut,i}^{\left(s\right)}\cdot P_{\text{Thrifty}}(Y|X,s,i)$$
  where ${ℐ}_{X,s}$ is defined as in (5).

Since both observed and Thrifty-predicted expected counts are calculated on the same phylogenetic trees and per-site mutation probabilities are small, branch lengths approximately cancel when taking ratios, allowing direct comparison of mutation counts rather than rates:

(10) $$\text{Rate}\;ratio(X\to Y|s,\;V\;\text{family})=\frac{{Count}_{\text{observed}}(X\;\to \;Y\;|\;s,\;V\;\text{family})}{{Count}_{\text{expected}}(X\;\to \;Y\;|\;s,\;V\;\text{family})}$$

We applied the same pseudocount smoothing (0.5) to both observed and expected counts and the same filtering criteria (expected mutations ≥ 5), then compared observed productive mutation counts to Thrifty-predicted expected counts. This approach retained 2877 out of 3247 testable substitutions for validation.

### Comparison to DASM selection factors

For both validation approaches, we compared log-transformed rate/count ratios to median log DASM selection factors, aggregated using the same filtering and summarization criteria described in “Entrenchment detection using DASM selection factors” above (depth 2 PCPs, germline-codon parents, single-nucleotide-reachable targets, ≥10 observations, median per combination). We assessed agreement using Pearson correlation coefficients and orthogonal regression (Deming, *λ* = 1), which is more appropriate than ordinary least squares regression when both variables have measurement error. To assess whether entrenched sites showed consistent purifying selection, we verified that substitutions identified as entrenched fell in the purifying selection region (log ratio *<* 0) of the validation plots.

## Supplementary Material

Supplement 1

Supplement 2

Supplement 3

## Figures and Tables

**Table 1:** Antibody repertoire datasets used in this study. PCP counts reflect all parent–child pairs in the source dataset. Individual analyses in this paper apply additional filters, including restriction to depth=2 parent–child pairs, as described in the relevant Methods subsections.

Dataset	Sequence type	Individuals	Clonal families	PCPs	Role in this study	Reference

Jaffe	Productive	4	57,364	228,790	DASM training dataset; here used for Thrifty-based validation of DASM selection factors (Approach 2).	[28]
Tang	Productive	21	146,920	522,586	DASM training dataset; here used for Thrifty-based validation of DASM selection factors (Approach 2).	[29, 30]
Rodriguez	Productive	50	7,769	21,754	Held-out test dataset (not used for DASM training); used for entrenchment inference from DASM selection factors and for out-of-frame-based validation (Approach 1).	[25]
Tang-SHM	Out-of-frame (non-productive)	21	3,209	9,984	Neutral baseline for validation (Approach 1); held-out test dataset for Thrifty, except for its multi-hit correction component (3 parameters) which was fit on these data.	[29, 30]
