# Supplementary material for: Entrenchment of germline amino-acid differences in antibody affinity maturation
Source: bioRxiv. 2026 May 23:2026.04.21.720000. Originally published 2026 Apr 23. Preprint. [Version 2] doi: 10.64898/2026.04.21.720000 (PMC13131580; doi:10.64898/2026.04.21.720000)
Supplement: 1 [file NIHPP2026.04.21.720000V2-supplement-1.pdf]

bioRxiv preprint doi: https://doi.org/10.64898/2026.04.21.720000; this version posted May 23, 2026. The copyright holder for this preprint (which was not certified by peer review) is the author/funder, who has granted bioRxiv a license to display the preprint in perpetuity. It is made available under a CC-BY 4.0 International license.

Supplementary Materials

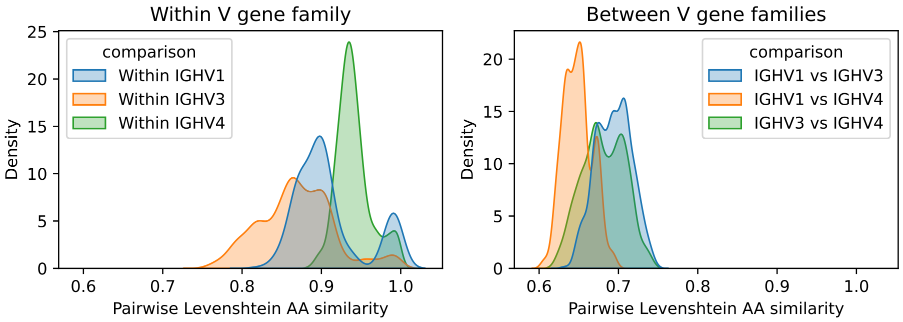

Figure S1: **Pairwise amino acid similarity between IGHV genes.** Kernel density estimates of pairwise Levenshtein amino acid similarity for IGHV1, IGHV3, and IGHV4. Top: within-family comparisons, where most gene pairs differ at up to ∼10% (IGHV4), ∼15% (IGHV1), or ∼20% (IGHV3). Bottom: between-family comparisons, where pairs differ at 25–40%.

bioRxiv preprint doi: https://doi.org/10.64898/2026.04.21.720000; this version posted May 23, 2026. The copyright holder for this preprint (which was not certified by peer review) is the author/funder, who has granted bioRxiv a license to display the preprint in perpetuity. It is made available under a CC-BY 4.0 International license.

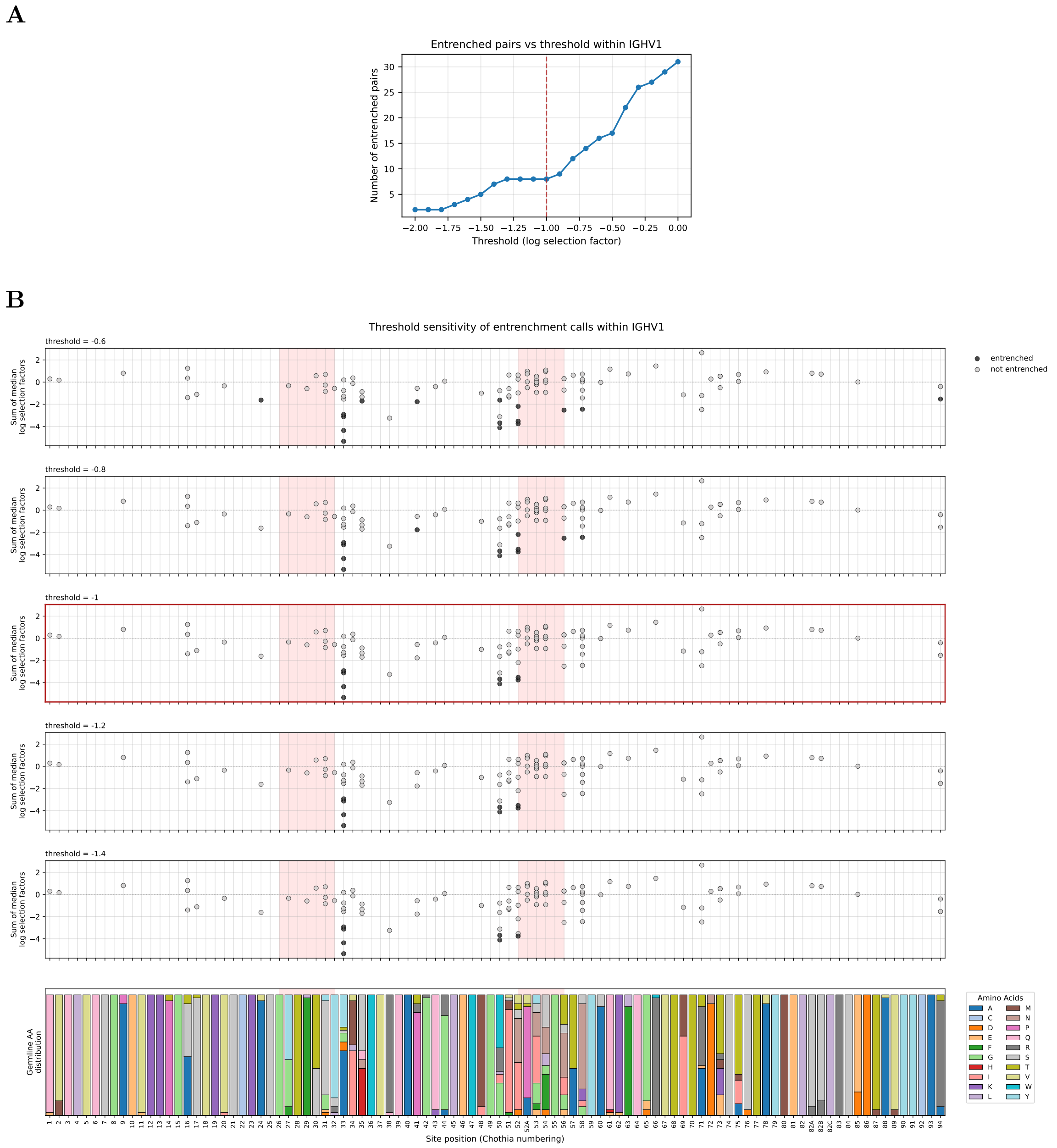

Figure S2: **Threshold sensitivity of entrenchment calls within IGHV1.** **(A)** Number of entrenched amino acid pairs as a function of the log selection factor threshold. The dashed red line marks the chosen cutoff of -1, at which we identify 8 entrenched pairs at 3 sites. Tightening to -1.4 retains 7 of these 8 pairs and all 3 sites. **(B)** Per-site view at five thresholds (top to bottom: -0.6, -0.8, -1, -1.2, -1.4). Filled points mark entrenched amino acid pairs, open points mark non-entrenched pairs; CDRs backgrounded in red. The bottom track shows the germline amino acid distribution across V genes at each site.

bioRxiv preprint doi: https://doi.org/10.64898/2026.04.21.720000; this version posted May 23, 2026. The copyright holder for this preprint (which was not certified by peer review) is the author/funder, who has granted bioRxiv a license to display the preprint in perpetuity. It is made available under a CC-BY 4.0 International license.

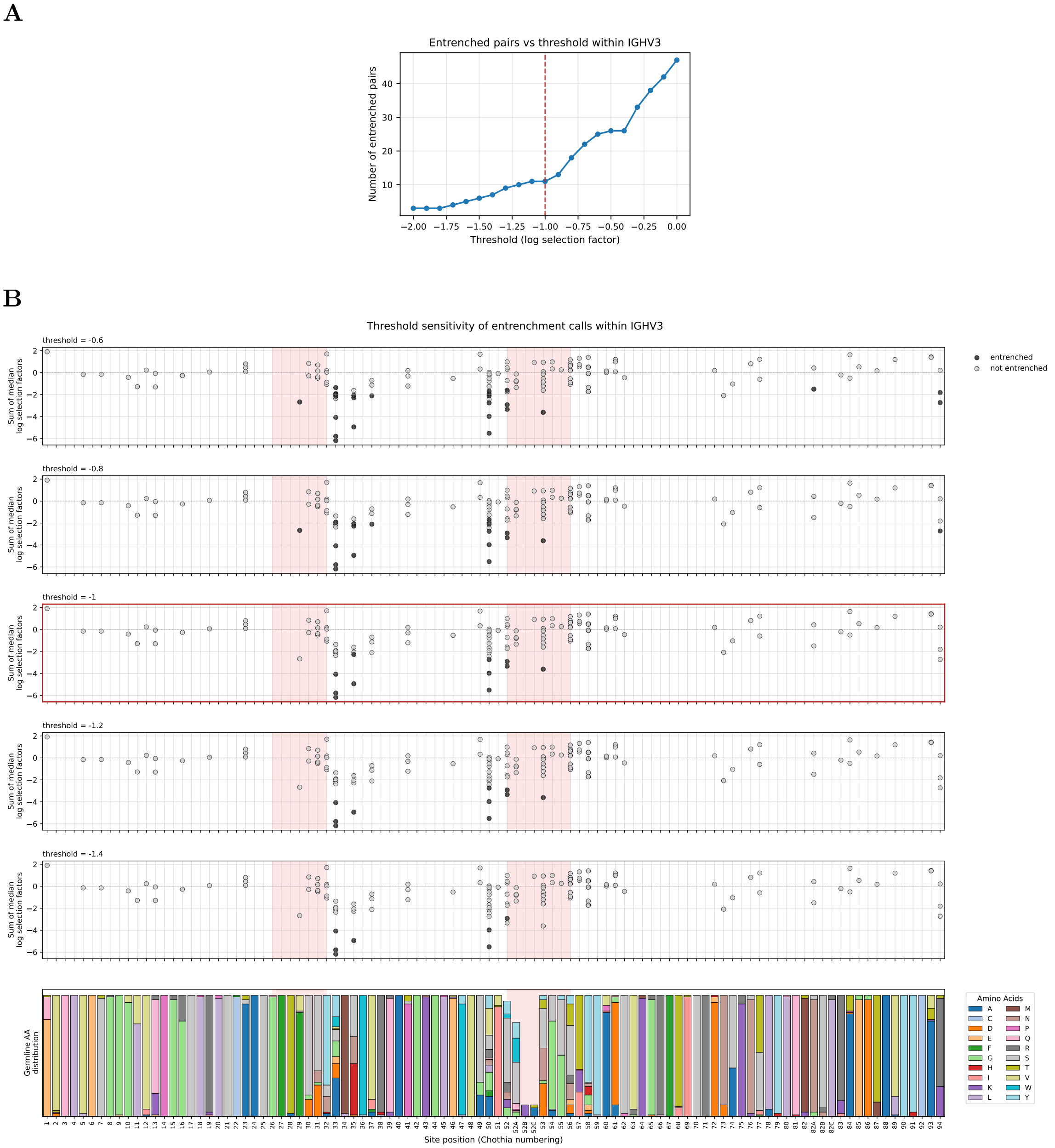

Figure S3: **Threshold sensitivity of entrenchment calls within IGHV3. (A)** Number of entrenched amino acid pairs as a function of the log selection factor threshold. The dashed red line marks the chosen cutoff of −1, at which we identify 11 entrenched pairs at 5 sites. Tightening to −1.4 retains 7 of these 11 pairs and 4 of the 5 sites. **(B)** As in Figure S2B, per-site view at five thresholds (top to bottom: −0.6, −0.8, −1, −1.2, −1.4).

bioRxiv preprint doi: https://doi.org/10.64898/2026.04.21.720000; this version posted May 23, 2026. The copyright holder for this preprint (which was not certified by peer review) is the author/funder, who has granted bioRxiv a license to display the preprint in perpetuity. It is made available under a CC-BY 4.0 International license.

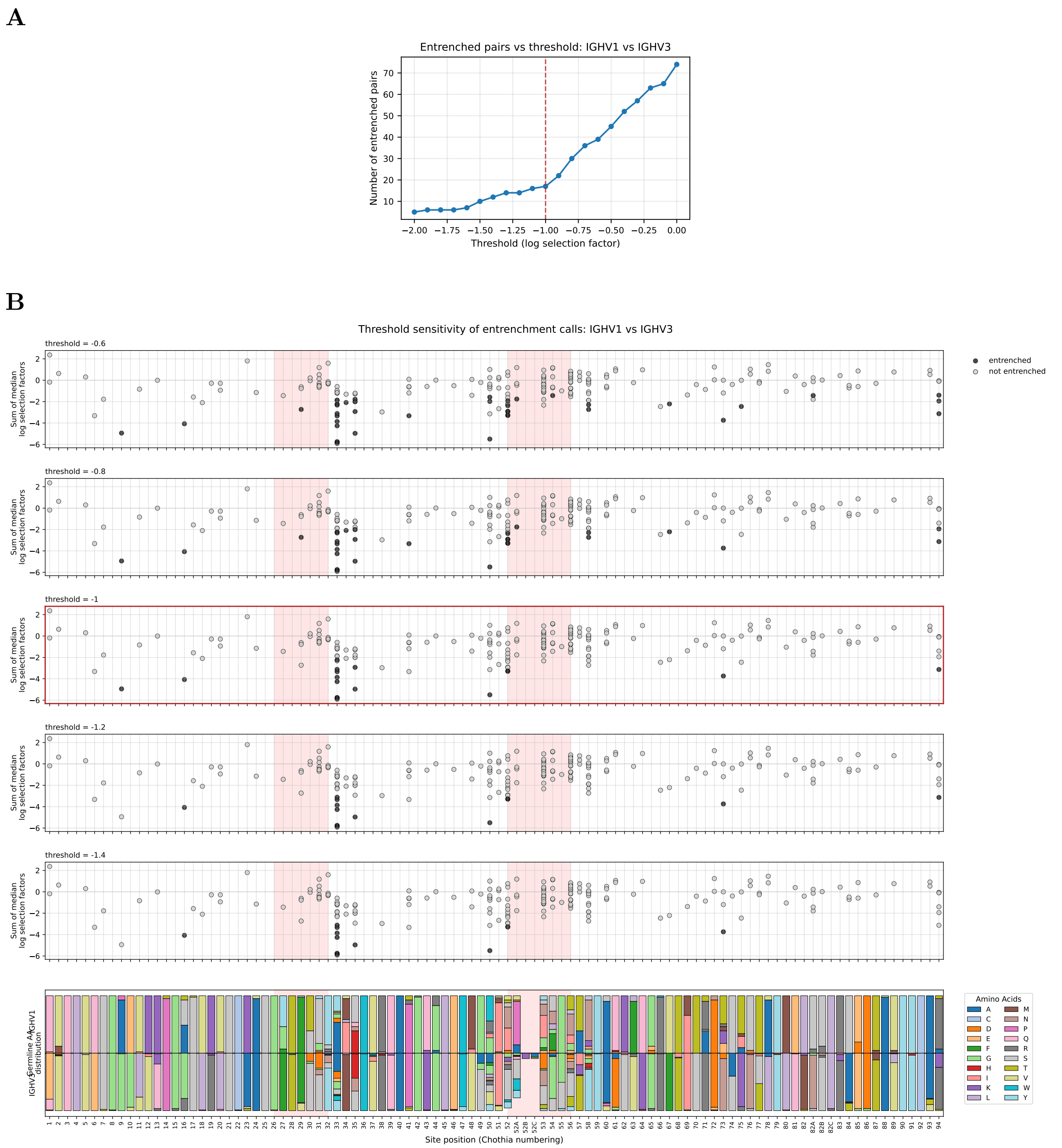

Figure S4: **Threshold sensitivity of entrenchment calls between IGHV1 and IGHV3.** **(A)** Number of entrenched amino acid pairs as a function of the log selection factor threshold. The dashed red line marks the chosen cutoff of -1, at which we identify 17 entrenched pairs at 8 sites. Tightening to -1.4 retains 12 of these 17 pairs and 6 of the 8 sites. **(B)** As in Figure S2B, per-site view at five thresholds (top to bottom: -0.6, -0.8, -1, -1.2, -1.4).

bioRxiv preprint doi: https://doi.org/10.64898/2026.04.21.720000; this version posted May 23, 2026. The copyright holder for this preprint (which was not certified by peer review) is the author/funder, who has granted bioRxiv a license to display the preprint in perpetuity. It is made available under a CC-BY 4.0 International license.

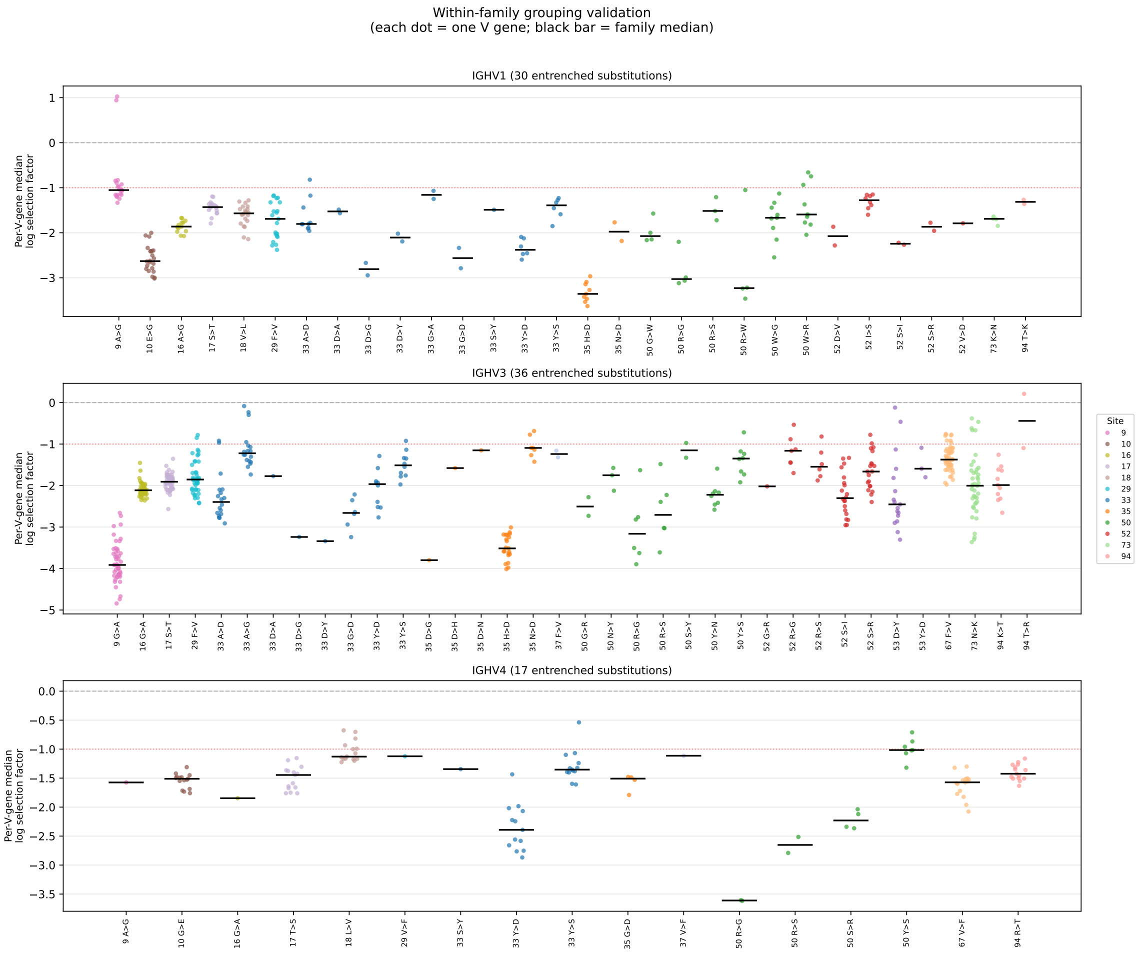

Figure S5: **Within-family grouping validation: per-V-gene-allele median selection factors at entrenched sites from both within-family and between-family analyses.** Each dot represents the median log selection factor for one V gene allele at one entrenched substitution, colored by site. Black bars show the family-level median used in the main analysis. The dashed red line indicates the -1 entrenchment threshold. The tight clustering of per-V-gene-allele medians around the family median confirms that pooling V gene alleles within a family does not create false entrenchment calls.

bioRxiv preprint doi: https://doi.org/10.64898/2026.04.21.720000; this version posted May 23, 2026. The copyright holder for this preprint (which was not certified by peer review) is the author/funder, who has granted bioRxiv a license to display the preprint in perpetuity. It is made available under a CC-BY 4.0 International license.

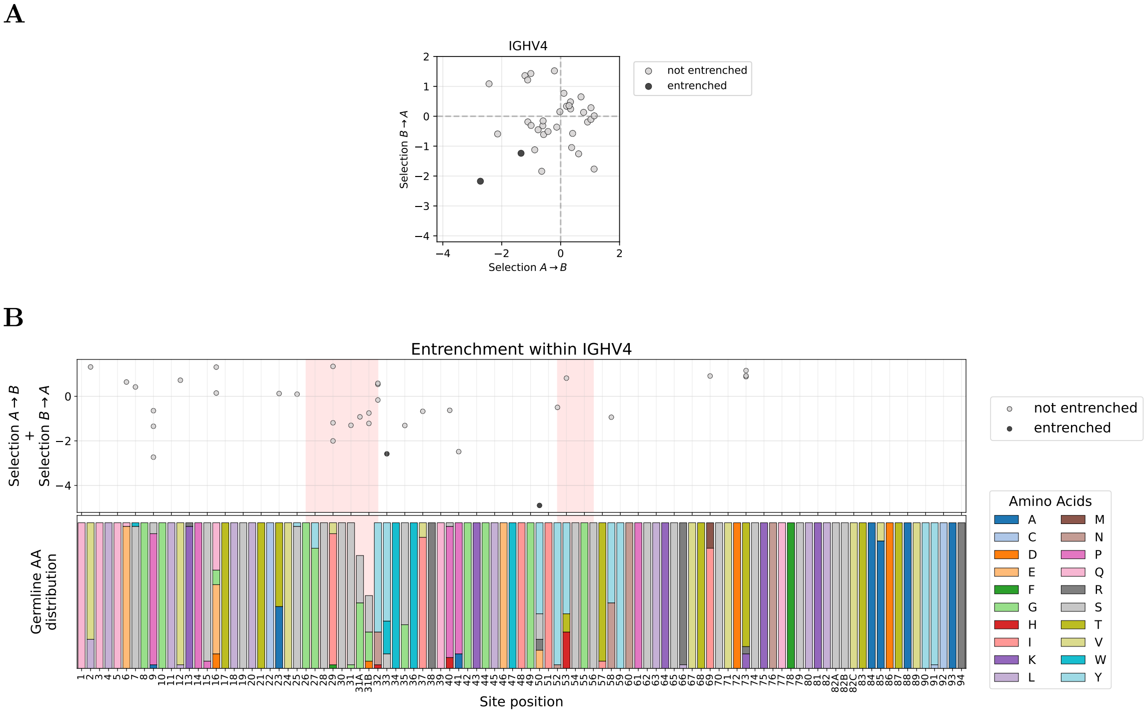

Figure S6: **Within-family entrenchment analysis for IGHV4.** **(A)** Reciprocal median log selection factors (selection $A \to B$ and selection $B \to A$) for amino acid pairs at sites where V genes within IGHV4 differ in germline identity; entrenched pairs (both reciprocal median log selection factors < -1) are highlighted. **(B)** Entrenchment mapped along the V gene sequence, analogous to Figure 2B. IGHV4 shows recurrent entrenched sites consistent with those identified in IGHV1 and IGHV3.

bioRxiv preprint doi: https://doi.org/10.64898/2026.04.21.720000; this version posted May 23, 2026. The copyright holder for this preprint (which was not certified by peer review) is the author/funder, who has granted bioRxiv a license to display the preprint in perpetuity. It is made available under a CC-BY 4.0 International license.

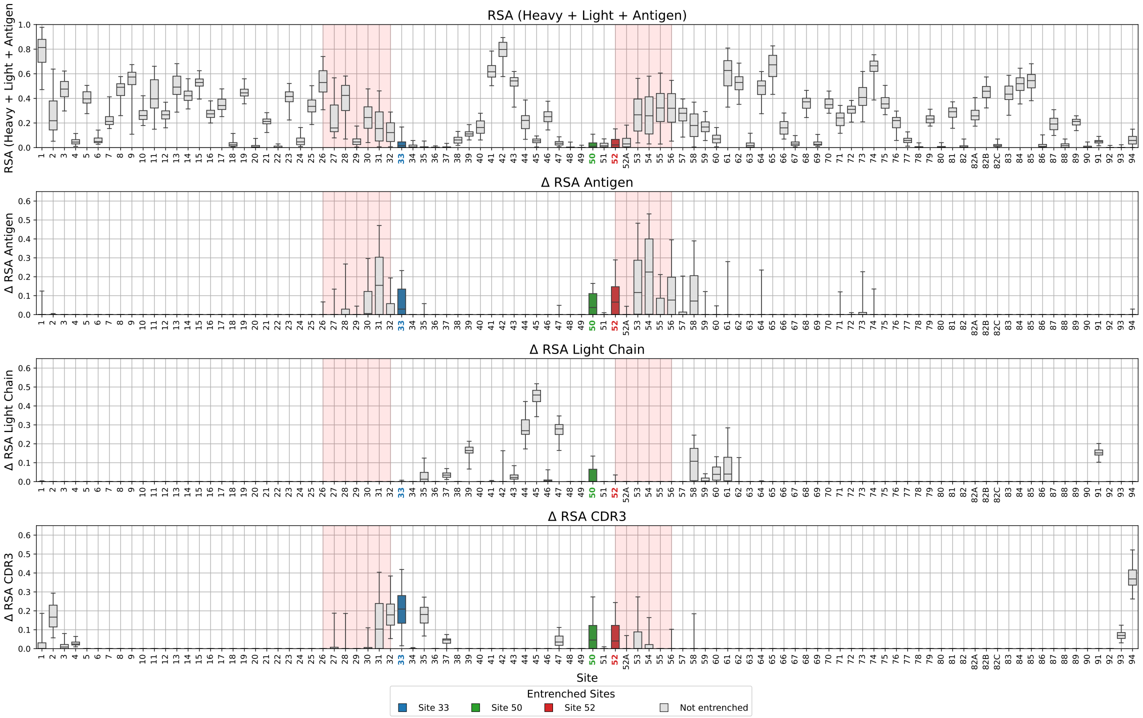

Figure S7: **RSA analysis for IGHV1 family at germline-encoded sites with entrenched amino acids within the V family.** Left: Relative solvent accessibility (RSA) in the full complex. Center: Change in RSA upon antigen removal (antigen effect). Right: Change in RSA upon light chain removal (light chain effect). This is the IGHV1 counterpart to the IGHV3 analysis shown in Figure 3D.

bioRxiv preprint doi: https://doi.org/10.64898/2026.04.21.720000; this version posted May 23, 2026. The copyright holder for this preprint (which was not certified by peer review) is the author/funder, who has granted bioRxiv a license to display the preprint in perpetuity. It is made available under a CC-BY 4.0 International license.

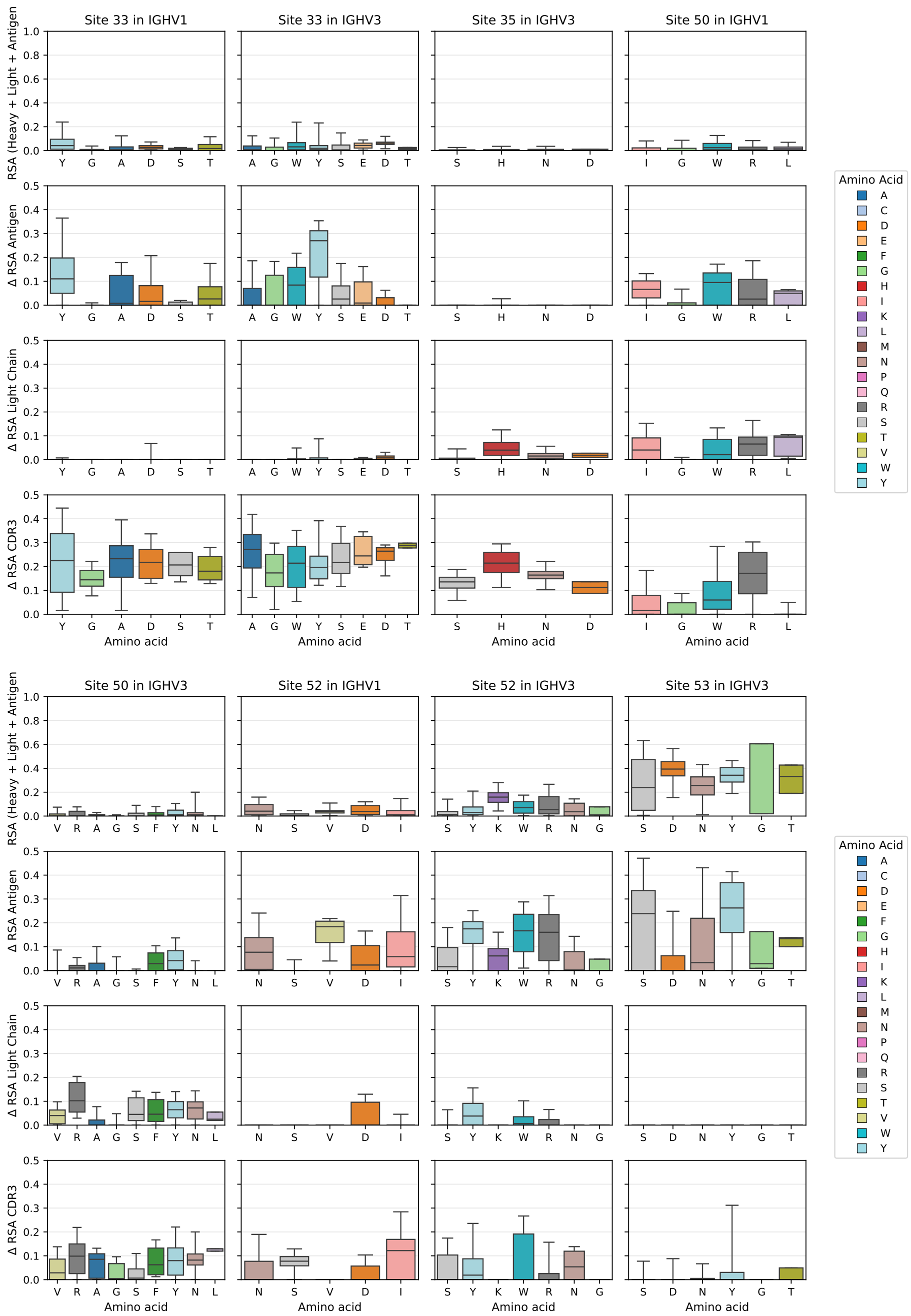

Figure S8: **RSA properties at entrenched sites by germline amino acid.** RSA properties for all within-family entrenched sites in IGHV1 and IGHV3. Row one shows RSA of the amino acid in the full heavy chain, light chain, and antigen complex. Row two shows the change in RSA upon antigen removal (antigen burial). Row three shows the change in RSA upon light chain removal (light chain burial). Row four shows the change in RSA upon CDR3 removal (positions 95–102).

bioRxiv preprint doi: https://doi.org/10.64898/2026.04.21.720000; this version posted May 23, 2026. The copyright holder for this preprint (which was not certified by peer review) is the author/funder, who has granted bioRxiv a license to display the preprint in perpetuity. It is made available under a CC-BY 4.0 International license.

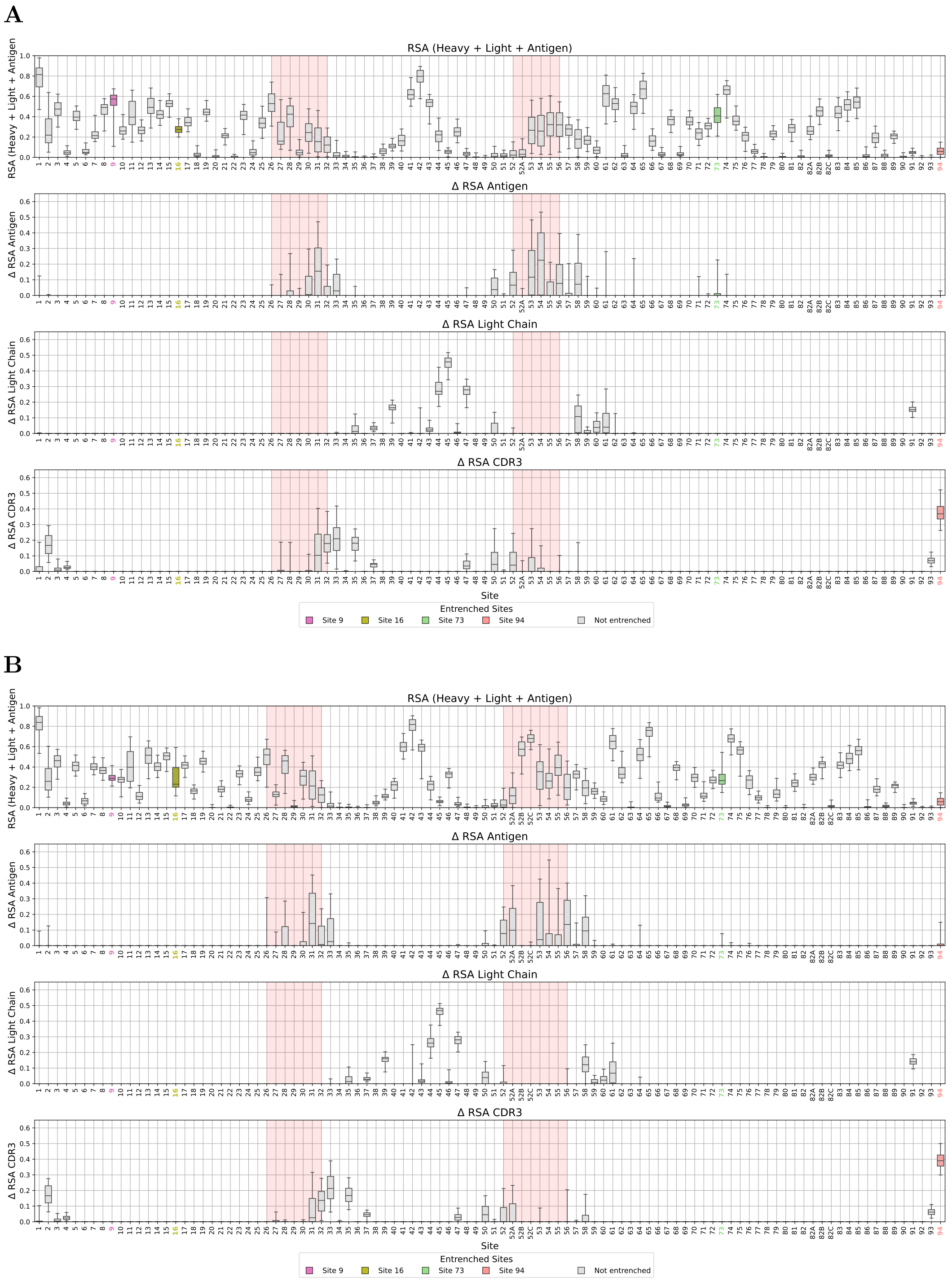

Figure S9: **RSA analysis at germline-encoded sites with amino acids entrenched between V families but not within V families (newly added sites). (A)** IGHV1 family: relative solvent accessibility (RSA) of sites in the full antibody-antigen complex, and change in RSA upon removal of antigen, light chain, or CDR3 (positions 95–102). **(B)** IGHV3 family: same analysis as (A).

bioRxiv preprint doi: https://doi.org/10.64898/2026.04.21.720000; this version posted May 23, 2026. The copyright holder for this preprint (which was not certified by peer review) is the author/funder, who has granted bioRxiv a license to display the preprint in perpetuity. It is made available under a CC-BY 4.0 International license.

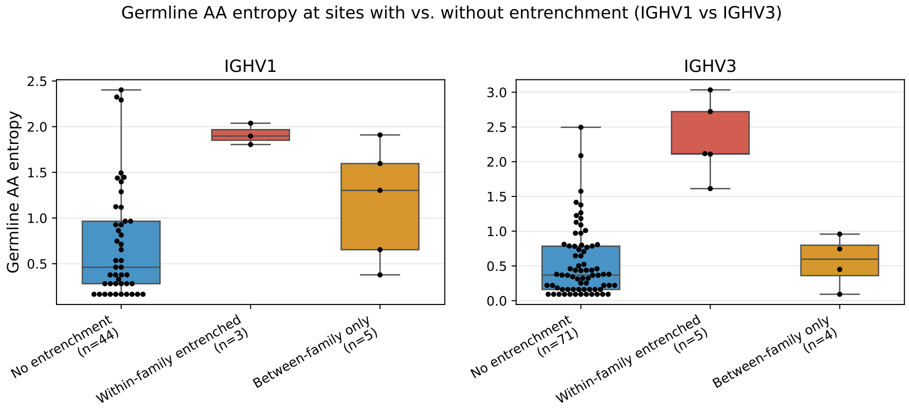

Figure S10: **Shannon entropy for sites entrenched within and between IGHV1 and IGHV3.** Germline diversity (Shannon entropy) at sites classified as non-entrenched, within-family entrenched, or between-family entrenched (excluding sites that overlap with within-family entrenched). Between-family-only entrenched sites show lower germline diversity than within-family entrenched sites, consistent with structural rather than diversifying constraints. Sites with only one germline amino acid across V genes in the family (entropy = 0) are excluded.

bioRxiv preprint doi: https://doi.org/10.64898/2026.04.21.720000; this version posted May 23, 2026. The copyright holder for this preprint (which was not certified by peer review) is the author/funder, who has granted bioRxiv a license to display the preprint in perpetuity. It is made available under a CC-BY 4.0 International license.

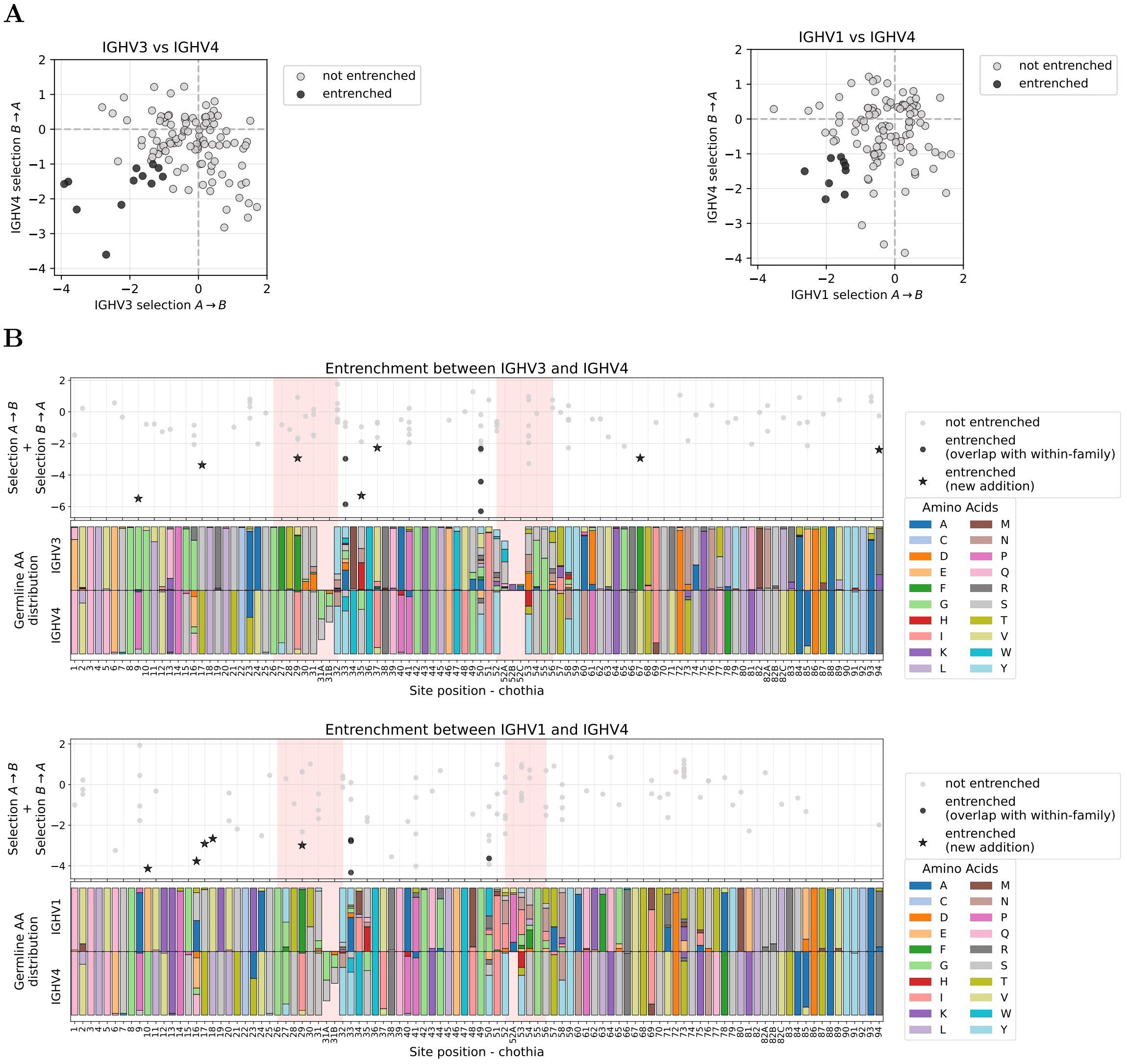

Figure S11: **Between-family entrenchment analysis for additional V-family pairs.** Panels show results for IGHV3 vs IGHV4 (left in A, top in B) and IGHV1 vs IGHV4 (right in A, bottom in B). **(A)** Reciprocal median log selection factors (selection $A \to B$ vs. selection $B \to A$) for amino acid pairs at sites where the two families differ in germline identity; entrenched pairs (both < -1) are highlighted. **(B)** Entrenchment mapped along the V gene sequence with within-family overlay, analogous to Figure 5B.

bioRxiv preprint doi: https://doi.org/10.64898/2026.04.21.720000; this version posted May 23, 2026. The copyright holder for this preprint (which was not certified by peer review) is the author/funder, who has granted bioRxiv a license to display the preprint in perpetuity. It is made available under a CC-BY 4.0 International license.

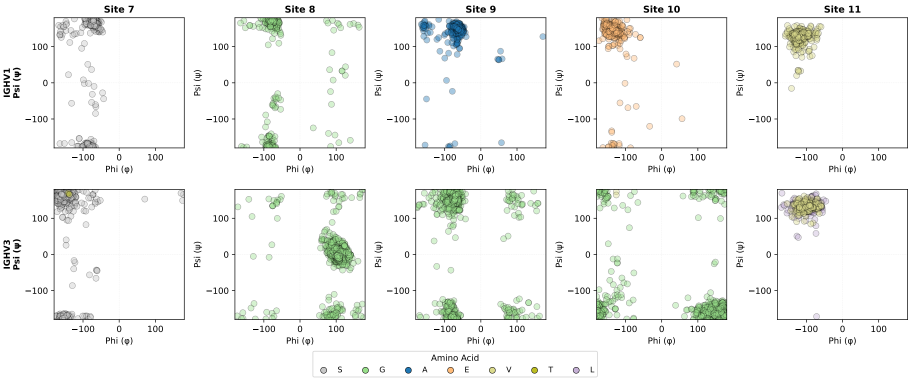

Figure S12: **Backbone data across SAbDab for entrenchment at site 9.** Ramachandran plots for sites 7–11 showing backbone dihedral angles (phi and psi) in IGHV1 (top) and IGHV3 (bottom) across SAbDab structures. The distinct angle distributions confirm family-specific backbone conformations at the site 9 region.

Table S1: **Structural analysis of hydrogen bonding networks at loop 73–75 in IGHV1 and IGHV3 Fab crystal structures.** Each row summarizes the local H-bond interactions observed at the loop containing entrenched site 73 for one crystal structure, derived from ChimeraX `hbonds` analysis with default geometric criteria (effective ∼3.5 Å donor–acceptor cutoff for N/O pairs). In IGHV1, K73 has no consistent framework hydrogen bond partner, while T75 is anchored by hydrogen bonds to both D72 and T77 in all four structures. In IGHV3, N73 is anchored by two contacts present in all four structures: a hydrogen bond from the R71 guanidinium and a donor hydrogen bond to a CDR-H2 backbone carbonyl (the acceptor residue varies across structures: H52A, N52A, G52A, or W53); the K75 sidechain is free of hydrogen bonds. Notation key: each H-bond is annotated as sidechain (sc) or backbone (bb) on each side (backbone = amide N and carbonyl O). Specific atom names appear in parentheses only to disambiguate multiple contacts on the same arginine: NE is the ε guanidinium nitrogen and NH1/NH2 are the two terminal guanidinium nitrogens. All are sidechain atoms.

IGHV1 Structures

PDB	Res. (Å)	71–75 Motif	Site 73 (K)	Site 74 (S)	Site 75 (T)
6VY4	2.00	ADKST	sc → CDR-H2 Pro bb (3.47 Å, chain H)	Free (chain H rotamer)	bb → D72 bb; sc → D72 bb; sc ↔ T77 sc; bb ← T77 sc
8G3Z	2.30	ADKST	Free	sc + bb → D72 sc	sc + bb → D72 sc; sc ↔ T77 sc; bb ← K23 sc
7X29	2.49	ADKST	sc → antigen bb	Free	sc + bb → D72 sc; bb ← S76 sc, T77 sc
2NY6	2.80	ADKST	Weak sc → D55 sc (3.51 Å, at the relaxed-distance limit)	sc → D72 sc	bb → D72 bb; sc → D72 sc; sc ↔ T77 sc; bb ← T77 sc

IGHV3 Structures

PDB	Res. (Å)	71–75 Motif	Site 73 (N)	Site 74 (S/A)	Site 75 (K)
4H8W	1.85	RDNSK	sc ← R71 sc (NE + NH2); sc → CDR-H2 N52A bb	S; sc + bb → D72 sc	sc free; bb → D72 bb + D72 sc; bb ← T77 sc
3BN9	2.17	RDNSK	sc ← R71 sc (NE + NH1); sc → CDR-H2 G52A bb	S; sc + bb → D72 sc	sc free; bb → D72 bb + D72 sc; bb ← T77 sc
6ULE	2.55	RDNSK	sc ← R71 sc (NH2); sc → CDR-H2 H52A bb	S; free	sc free; bb → D72 bb
6PPG	2.75	RDNAK	sc ← R71 sc (NE + NH1); sc → CDR-H2 W53 bb	A (no sidechain hydroxyl)	sc free; bb → D72 bb + D72 sc; bb ← S77 sc

bioRxiv preprint doi: https://doi.org/10.64898/2026.04.21.720000; this version posted May 23, 2026. The copyright holder for this preprint (which was not certified by peer review) is the author/funder, who has granted bioRxiv a license to display the preprint in perpetuity. It is made available under a CC-BY 4.0 International license.

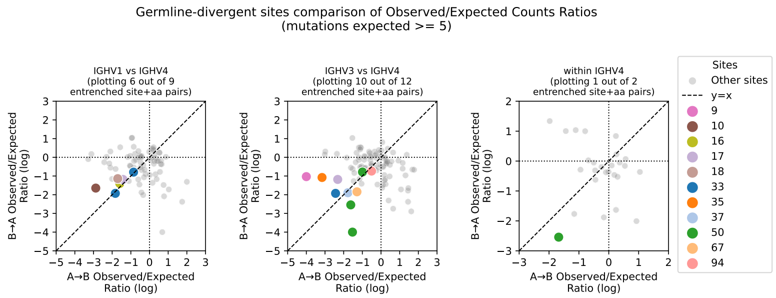

Figure S13: **Pairwise validation of entrenched substitutions for IGHV4 comparisons.** Analogous to Figure 7C but for within-IGHV4, IGHV1 vs IGHV4, and IGHV3 vs IGHV4 comparisons. Of the 23 entrenched reciprocal pairs involving IGHV4, 17 (74%) had sufficient data in both directions. All 17 showed purifying selection in both directions, with all falling below -0.5 and 14 (82%) below the -1 entrenchment threshold.
